# Inference of Population Splits and Mixtures from Genome-Wide Allele Frequency Data

**DOI:** 10.1371/journal.pgen.1002967

**Published:** 2012-11-15

**Authors:** Joseph K. Pickrell, Jonathan K. Pritchard

**Affiliations:** 1 Department of Human Genetics, University of Chicago, Chicago, Illinois, United States of America; 2 Howard Hughes Medical Institute, University of Chicago, Chicago, Illinois, United States of America; Stanford University, United States of America

## Abstract

Many aspects of the historical relationships between populations in a species are reflected in genetic data. Inferring these relationships from genetic data, however, remains a challenging task. In this paper, we present a statistical model for inferring the patterns of population splits and mixtures in multiple populations. In our model, the sampled populations in a species are related to their common ancestor through a graph of ancestral populations. Using genome-wide allele frequency data and a Gaussian approximation to genetic drift, we infer the structure of this graph. We applied this method to a set of 55 human populations and a set of 82 dog breeds and wild canids. In both species, we show that a simple bifurcating tree does not fully describe the data; in contrast, we infer many migration events. While some of the migration events that we find have been detected previously, many have not. For example, in the human data, we infer that Cambodians trace approximately 16% of their ancestry to a population ancestral to other extant East Asian populations. In the dog data, we infer that both the boxer and basenji trace a considerable fraction of their ancestry (9% and 25%, respectively) to wolves subsequent to domestication and that East Asian toy breeds (the Shih Tzu and the Pekingese) result from admixture between modern toy breeds and “ancient” Asian breeds. Software implementing the model described here, called *TreeMix*, is available at http://treemix.googlecode.com.

## Author Summary

With modern genotyping technology, it is now possible to obtain large amounts of genetic data from many populations in a species. An important question that can be addressed with these data is: what is the history of these populations? There is a long history in population genetics of inferring the relationships among populations as a bifurcating tree, analogous to phylogenetic trees for representing the evolution of species. However, it has long been recognized that, since populations from the same species exchange genes, simple bifurcating trees may be an incorrect representation of population histories. We have developed a method to address this issue, using a model which allows for both population splits and gene flow. In application to humans, we show that we are able to identify a number of both previously known and unknown episodes of gene flow in history, including gene flow into Cambodia of a population only distantly related to modern East Asia. In application to dogs, we show that the boxer and basenji breeds have a considerable component of ancestry from grey wolves subsequent to domestication.

## Introduction

The extant populations in a species result from an often-complex demographic history, involving population splits, gene flow, and changes in population size. It has long been recognized that genetic data can be used to learn about this history [1]–[3]. In humans, early approaches to inferring history from genetics were limited to using a relatively small number of blood group or other protein polymorphisms [1], [4]–[6]. These types of studies were then superseded by analyses of DNA markers, which have progressed from single marker studies [3] to studies involving hundreds of thousands of markers [7]. It is now feasible to collect genome-wide genetic data in any species; to a large extent it is no longer the data collection, but rather the statistical models used for analysis, that limit the historical insight possible.

There are many statistical approaches to demographic inference from genetic data. One approach is to develop an explicit population genetic model for the history of a set of populations, framed in terms of the effective population sizes of the populations, the times of population splits, the times of demographic events (such as population bottlenecks), and other relevant parameters. The values of these parameters can then be learned from the data using a variety of techniques, often involving simulation [8]–[16]. These approaches have the advantage of allowing flexible modeling of a wide variety of demographic scenarios, but the disadvantage that they can only be applied to one or a few populations at a time.

Another type of approach to learning about population history uses methods that summarize the major components of genetic variation in a sample by clustering or principal components analysis [17]–[20]. Although these methods do not model history explicitly, the inferred components can often be interpreted *post hoc* as representing historical populations, and individuals or populations that are mixtures of different components as evidence of admixture between these populations (e.g., [17], [21]–[23]). However, these methods are not directly informative about history; indeed, the relationship between the major components of genetic variation and true underlying demography is not always intuitive [24]–[26].

A different class of approaches focuses on the relationships between populations, by representing a set of populations as a bifurcating tree [1], [27]–[32]. In these models, the details of the demographic histories of the population are absorbed into the branch lengths of the tree [1], [33]. This approach has the advantage of being applicable to large numbers of populations; however, a major caveat when modeling the history of populations as a tree is that gene flow violates the assumptions of the model [2], [34], [35]. It is often difficult to know, *a priori*, how well the history fits a simple bifurcating tree. Explicit tests for the violation of a tree model have been developed [35]–[40]. These tests have been used, most notably, to infer the existence of gene flow between modern and archaic humans [39], [41], [42], as well as between diverged modern human populations [37], [43], [44].

In this paper, we present a unified statistical framework for building population trees and testing for the presence of gene flow between diverged populations. In this framework, the relationship between populations is represented as a graph, allowing us to model both population splits and gene flow. Graph-based models are of growing interest in phylogenetics [45], [46], but have been rarely used in population genetics (with some exceptions [37], [40], [47]).

## Results

The starting point for our model was first proposed by Cavalli-Sforza and Edwards [1], and we draw additionally on related models by Nicholson et al. [33] and Coop et al. [48]. Our goal is to provide a statistical framework for inferring population networks that is motivated by an explicit population genetic model, but sufficiently abstract to be computationally feasible for genome-wide data from many populations (say, 10–100). Our primary aim is to represent the topology of relationships between populations, rather than the precise times of demographic events.

Our approach to this problem is to first build a maximum likelihood tree of populations. We then identify populations that are poor fits to the tree model, and model migration events involving these populations. Below, we first describe this approach in an idealized setting, and then describe the modifications necessary for implementation in practice.

### Model

In the most simple case, consider a single SNP, and let the allele frequency of one of the alleles at this SNP in an ancestral population be 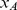. (We use a lowercase 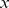 to denote that this is a parameter rather than a random variable. We initially consider distributions conditional on 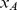). Now consider a descendant population 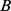. We model 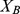, the allele frequency of the SNP in population 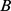, as:
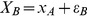
(1)with
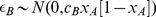
(2)where 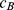 is a factor that reflects the amount of genetic drift that has occurred between the ancestral population and 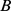. This Gaussian model was first introduced by Cavalli-Sforza and Edwards [1], and the motivation for this model is outlined in Nicholson et al. [33], if the amount of genetic drift between the two populations is small (at most on a timescale of the same order as the effective population size), then the diffusion approximation to a Wright-Fisher model of genetic drift leads to Equation 2 with 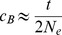, where 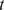 is the number of generations separating the two populations, and 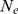 is the effective population size [33]. We do not model the boundaries of the allele frequencies at zero and one, nor do we consider new mutations. This means that this model will be most accurate for alleles that were at intermediate frequency in the ancestral population.

Now consider a descendant population of 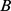; let us call this population 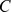, and the allele frequency in the population 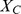. Using the same model:
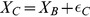
(3)

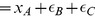
(4)where
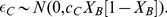
(5)


We can write down the expectation and variance of 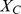 as:
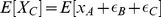
(6)

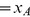
(7)and:
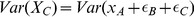
(8)

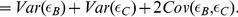
(9)We then assume that the amount of genetic drift between all the populations is small. This implies that 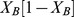 is well-approximated by 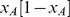 in Equation 5, and hence the amount of genetic drift between 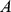 and 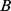 is approximately independent of the amount of genetic drift between 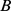 and 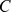 [35]. With these simplifications:
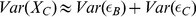
(10)

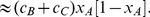
(11)We thus have a model for 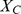, conditional on 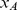:
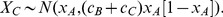
(12)


#### Multiple populations

**Figure 1:**
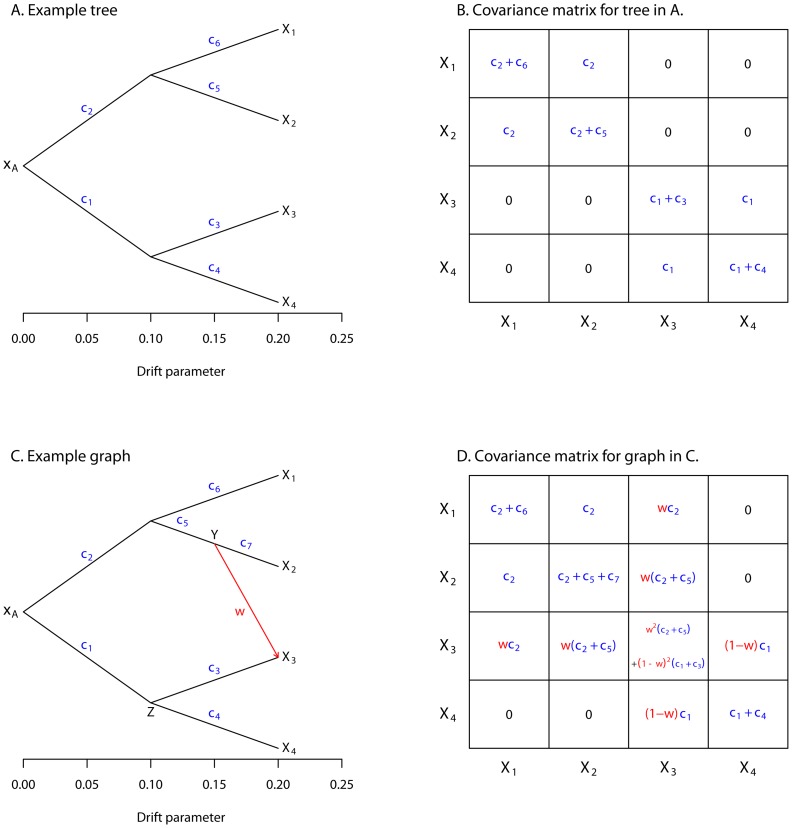
Simple examples. A. An example tree. B. The covariance matrix implied by the tree structure in A. Note that the covariance here is with respect to the allele frequency at the root, and that each entry has been divided by 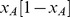 to simplify the presentation. C. An example graph. The migration edge is colored red. Parental populations for population 3 are labeled 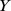 and 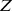; see the main text for details. D. The covariance matrix implied by the graph in C; again, each entry has been divided by 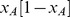. The migration terms are in red, and the non-migration terms are in blue.

Now consider a set of four populations, all related to an ancestral population by a tree, as depicted in Figure 1A. Let the allele frequencies in the four populations be denoted 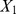, 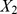, 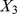, and 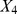, respectively, and the vector of all four frequencies be 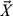. We want to write down a joint distribution for 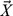 given the tree. We start by writing down the covariance between any two populations with respect to the ancestral allele frequency (i.e. 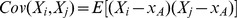). This is simply the variance of the common ancestor of the two populations:
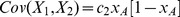
(13)

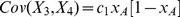
(14)

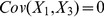
(15)and so on (Figure 1B).

Let us use 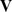 to denote the variance-covariance matrix of allele frequencies between populations implied by the tree. Now, if we knew the value of 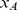, we could model 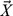 as:
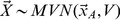
(16)where 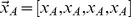 and 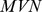 denotes the multivariate normal distribution. The covariance matrix 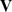 is distinct from the sample covariance matrix; we return to this distinction later. This multivariate normal model was proposed by [28]. Additionally, a multivariate normal model was used by Coop et al. [48] and Weir and Hill [49], although these authors did not explicitly model the variance-covariance matrix in terms of a tree.

#### Modeling migration

To extend this framework to include migration, we allow populations to have ancestry from multiple parental populations [35], [40]. The contribution of each parental population is weighted; if we assume admixture occurs in a single generation, these weights are related to the fraction of alleles in the descendant population that originated in each parental population. Consider population 3 in Figure 1C (recall that the allele frequency in this population is 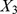). We have labeled the two parental populations 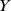 and 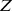; let the allele frequencies in these populations be 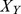 and 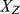, respectively. We model 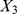 as:
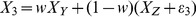
(17)where 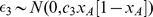. Note that there is some question as to how to weight 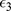, the genetic drift specific to population 3. In reality, it comes from three sources: drift since 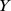 but before the population mixture, drift since 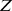 but before the population mixture, and drift since the mixture. These three components should have weights 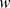, 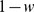, and 1, respectively. However, the three components are not all separately identifiable. For ease of computation, we estimate only a single drift parameter in this situation, and weight it by 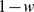 (Text S1, Figure S1).

Additionally, there is a choice of whether the edge from 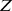 or the edge from 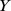 should be considered the “migration” edge; these two possibilities not identifiable in the model. In practice, we set the edge with the largest weight as the non-migration edge, and the other edge (or edges) as the “migration” edge(s).

With these simplifications, the variance of 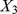 can be written in the mixture case as:
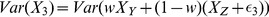
(18)

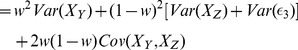
(19)We can now consider multiple populations related by a graph instead of a tree (Figure 1C). The variance-covariance matrix 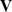 can be filled in as before, but now including terms for migration (Figure 1D). This model can be written in terms of a directed acyclic graph (the lack of cycles follows from the fact that no population can contribute genetic material to its own ancestor), where the 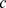 parameters correspond to edge lengths (Text S1). For subsets of up to four populations, this model is closely related to the “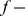 statistics” used as tests for treeness by Reich et al. [37] (Text S1).

#### Normalization

As described above, 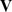 depends on the ancestral allele frequency 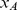. This means that the true variance-covariance matrix will differ by a scaling factor between SNPs with different values of 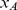. In much work on this type of model, investigators have normalized allele frequencies to account for this. One potential normalization is the arcsine square-root transformation [1]. However, a drawback to this normalization is that it is non-linear; the expected value of the allele frequency in the descendant populations is no longer 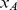, but pushed towards the boundaries, which could induce spurious correlations between the most drifted populations [50]. Another plausible transformation would be to scale all allele frequencies by 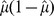, where 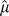 is the mean allele frequency across populations [19], [33]. Both of these transformations increase the influence of polymorphisms that were rare in the ancestral population. However, these are precisely the loci where the approximation of our model to a true population genetics model is most likely to break down. For these reasons, we choose to work directly with untransformed allele frequencies.

#### Properties of the sample covariance

In practice, the multivariate normal model in Equation 16 is impractical because we do not know the ancestral values of allele frequencies, but instead only the values in sampled descendant populations. This means that 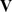 cannot be estimated directly from data. However, consider instead the *sample* covariance matrix 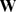, where 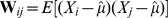, where 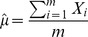, 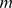 is the number of populations, and 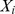 and 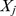 are the sample allele frequencies in populations 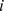 and 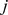. 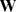 is closely related to 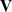 as follows:
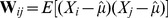
(20)

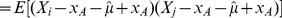
(21)

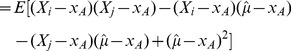
(22)

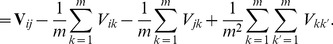
(23)In the following section, we will describe how we perform inference based on the sample covariance matrix 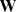.

#### Estimation from finite samples and many SNPs

Now assume that we have genotyped 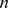 SNPs in each of 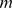 populations. Our goal is to use these data to estimate the population history graph 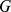: that is, 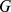 is a rooted, directed, acyclic graph with specified branch lengths and mixture weights, as described above and in Text S1. Let the sample allele frequency at SNP 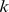 in population 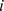 be 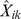. We can estimate the sample covariance matrix 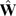:
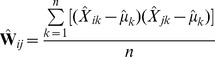
(24)where 
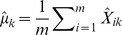
. The fact that in practice we have finite samples from each population and a finite number of SNPs has two effects on this matrix. First, sampling leads to a biased estimation of the terms on the diagonal, since sampling can be thought of as adding an amount of “drift” to each population (as well as smaller effects on the off-diagonal terms; see Text S1 for details). Additionally, it leads to some uncertainty in all terms. To account for the biased diagonal terms, in practice we calculate a corrected version of 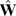 that removes this bias (Text S1). To account for uncertainty in the values of this matrix, we use a block resampling approach (see below). Finally, with multiple SNPs, we are working with SNPs with many different values of 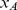. In this case, the 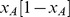 terms described above can be thought of as 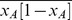; i.e., the mean across SNPs of 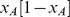.

We now want to write down a likelihood for 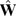 given 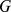 (which in turn implies the expected sample covariance matrix 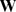). One possibility would be to use the Wishart distribution, since the sample covariance matrix of multivariate normal random variables has this form. However, computation of the Wishart density involves computationally-intensive matrix inversion, so we took an alternative approach. Consider the observed covariance between populations 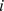 and 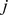, 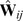. If we had a large number of independent genomic regions and estimated this quantity separately in each independent region, the sampling distribution would be approximately normal with mean 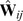 (by appeal to the central limit theorem). We thus model 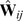 as:
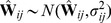
(25)where 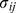 is the standard error in the estimation of 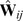. Because the allele frequencies at nearby SNPs are correlated (i.e., there is linkage disequilibrium), a naive estimate of 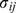 that treated each SNP as independent would be too small. We instead take a resampling approach to estimate 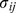. We split the genome into 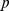 blocks, such that there are 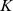 SNPs per block (with 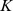 chosen so that the block sizes are larger than blocks of linkage disequilibrium) [36]. (If 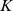 does not divide evenly into 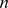, we discard the remaining SNPs.) We then calculate 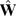 separately in each block. Let 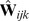 be the sample covariance between two populations 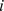 and 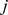 in block 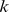. Now,
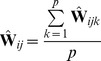
(26)and
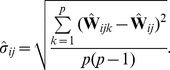
(27)If we take each pair of populations in turn, we can write down a composite likelihood for 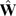:
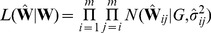
(28)where 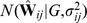 is a Gaussian density with mean 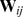 (computed from 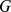 by Equation 23) and variance 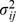 evaluated at 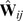.

#### Measuring model fit

Finally, we wanted to define measures for how well the model fits the data. First, we define the matrix of residuals in this model, 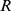. These quantities are useful for visualization and fitting:
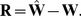
(29)Positive residuals indicate pairs of populations where the model underestimates the observed covariance, and thus populations where the fit might be improved by adding additional edges. Negative residuals indicate pairs of populations where the model overestimates the observed covariance; these are a necessary outcome of having positive residuals, but can also sometimes be interpreted as populations that are forced too close together due to unmodeled migration elsewhere in the graph. These residuals can be used to define a measure of the fraction of the variance in 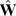 that is explained by 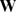. Let us call this fraction 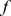:
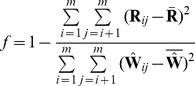
(30)where 
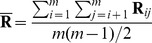
 and 
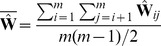
. This quantity approximates the fraction of the variance in relatedness between populations that is accounted for by the model.

#### Implementation

We implemented an algorithm, called *TreeMix*, that searches for the graph that maximizes the composite likelihood in Equation 28. A search that enumerates all graphs is infeasible unless 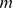 is very small, so to simplify the search we make the assumption that the history of the sampled populations is approximately tree-like. We thus start by searching for the maximum likelihood tree, taking an algorithmic approach similar to Felsenstein [30].

After building the tree, we fix the position of the root. (In the tree model the position of the root is not identifiable, as the evolution of allele frequencies along the tree is reversible under the Gaussian model when drift is assumed to be small. In a graph model, though the position of the root is partially identifiable, in all applications we assume that the position of the root is fixed using prior information about known outgroups). We then calculate the residual covariance matrix, 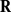, and add migration edges in a directed matter. First, we find the 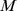 pairs of populations with the maximum residuals. We then attempt adding a migration edge between populations in the vicinity of each of the 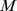 population pairs. For each attempted graph (or tree) topology, we optimize the branch lengths and migration edge weights (Methods).

After finding the single migration edge that most increases the likelihood, we attempt a series of local changes to the graph structure (Methods). We then iterate over this procedure to add additional migration edges. In principle, migration edges could be added until they are no longer statistically significant (see the following paragraph). In our experience, however, we prefer to stop adding migration events well before this point so that the resulting graph remains interpretable.

#### Significance testing

After building the maximum likelihood graph, we would like to quantify our uncertainty in the resulting graph structure. In particular, we would like to quantify our confidence in individual migration events. However, because the likelihood in Equation 28 is a composite likelihood, it cannot be used directly for formal tests for significance. Instead, we take a resampling approach to test the support for individual migration edges.

Consider a given migration edge, with corresponding weight 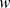. We wish to calculate a p-value for this weight (under the null hypothesis that 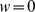, and for a fixed graph structure). To do this, we use the Wald statistic 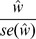, where 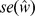 is the standard error in the estimate of the weight, which is distributed 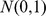 under the null. To obtain the standard error, recall that we have split the genome into 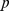 independent blocks. We use the jackknife estimates of both 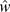 and the standard error in 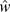 (where we jackknife over blocks). Let 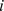 index blocks, and 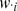 be the estimated weight computed using all blocks *except* 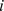. Then:
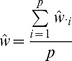
(31)

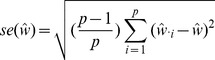
(32)This allows us to calculate a p-value for the migration edge. There are a number of complications to the interpretation of this p-value. First, there is the issue of multiple testing–there are at least 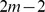 edges in the graph (recall that 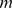 is the number of populations), and thus around 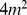 possible migration events. More importantly, the p-value is generated under a heavily parameterized model: we are comparing a fixed graph structure with a migration event to that same graph without the migration event. A “significant” p-value simply indicates that the hypothesized migration event significantly improves the fit to the data; this does not account for the possibility of errors in the graph structure, or indicate that the particular migration event tested is the correct one (rather than a migration event between a different pair of populations). For this reason, we treat the precise p-value generated by this procedure with caution, and use additional, less-parameterized methods like three- and four-population tests [37] to test the robustness of the inference.

### Simulations

**Figure 2:**
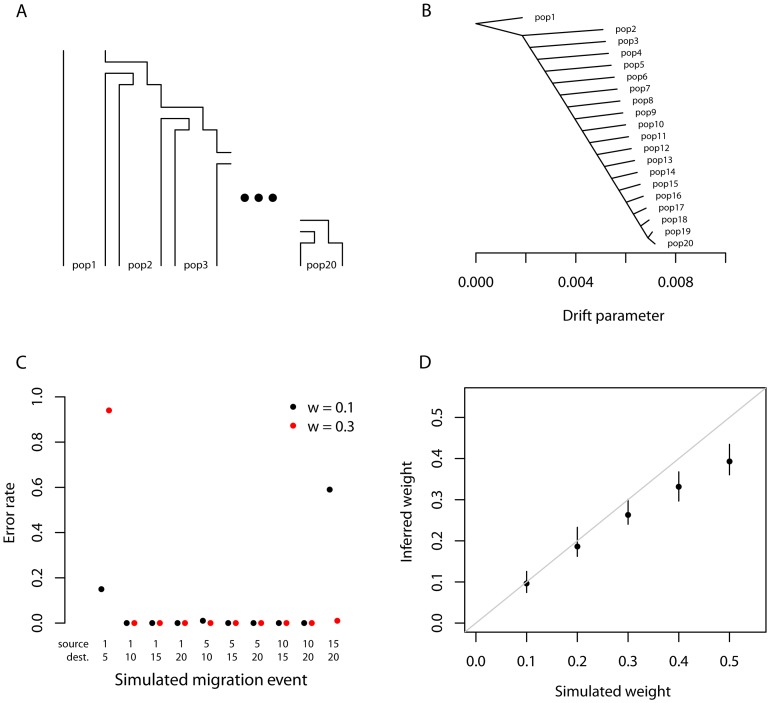
Performance on simulated data. A. The basic outline of the demographic model used. B. Trees inferred by *TreeMix*. We simulated 100 independent data sets, under the demographic model in A., and inferred the tree. All simulations gave the same topology; plotted are the mean branch lengths. C. Performance in the presence of migration. We added migration events to the tree in A. and inferred the structure of the graph. Each point represents the error rate over 100 independent simulations, defined as the fraction of simulations where the inferred graph topology does not perfectly match the simulated topology. On the x-axis we show the populations involved in the simulated migration event; e.g., if the source population is 1 and the destination population is 10, this is a migration event from population 1 to population 10, as labeled in A. D. Admixture weight estimation. We simulated admixture events with different weights from population 1 to population 10, and inferred the weight. Each point is the mean across 100 simulations, and the bar represents the range.

We tested the performance of the *TreeMix* method in simulations. We generated coalescent simulations from several histories; the basic structure was a set of 20 populations produced by a serial bottleneck model like that used by DeGiorgio et al. [51] to model human history (Figure 2A). The parameters of the simulations were chosen to be reasonable for non-African human populations; we used an effective population size of 10,000, and a history where all 20 populations share a common ancestor 2000 generations in the past. Each individual simulation involved 400 regions of approximately 500 kb each, and thus recapitulated many aspects of real data, including hundreds of thousands of loci and the presence of linkage disequilibrium.

#### Tree simulations

First, we tested the performance of the algorithm on truly tree-like data. We generated 100 independent simulations of 20 chromosomes from each population using the above demographic model without migration, and inferred population trees. The inferred trees perfectly matched the simulated model in all cases (Figure 2B, Figure S2), and the fitted tree model accounted for an average of 99.8% of the variance in 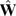. To test the effect of SNP ascertainment, we then inferred trees using only SNPs that were polymorphic in one of the populations (either population 1 or population 20); this ascertainment scheme did not decrease accuracy of the inferred topology, though it did alter the inferred branch lengths (Figure S3).

We used these simulations without migration to test the calibration of our p-values for migration events. For each simulation, after building the maximum likelihood tree, we introduced a migration event between two random populations and tested it for significance. As expected if the p-values are properly calibrated, their distribution is approximately uniform (Figure S4).

Finally, we performed tree simulations in a situation where fixed differences and new mutations (rather than shared polymorphisms inherited from a common ancestor) were common between the populations; in this context the population genetic interpretation of the model breaks down. We performed simulations where all the true branch lengths were 50 times longer than in the original model, corresponding to a history where the 20 populations share a common ancestor approximately 100,000 generations in the past. Again, we see no errors in the topology of the inferred trees (Figures S5, S6). In this situation, the covariances between closely-related populations tend to be slightly underestimated; in more extreme situations this could lead to spurious inferences of migration (Figures S5, S6). However, overall, these simulations suggest that the model will still be useful even in situations where the population genetic interpretation is not strictly correct.

#### Simulations with migration

We then introduced migration events into our simulations. We generated simulations under the same model described above; however, we now simulated an admixture event approximately 100 generations before the present where one population receives a fraction of its ancestry (either 10% or 30%) from one of the other populations. We tried ten different pairwise combinations of populations, and generated 100 simulations for each pair. For each simulation, we ran *TreeMix* and allowed it to infer a migration event. We then judged the error rate of the algorithm as the fraction of times the inferred topology of the graph was not exactly correct (this is a conservative estimate of the error rate, in that inferred graph topologies that are very close to the truth are counted as errors). In general, *TreeMix* was able to correctly infer the graph structure in these simulations (Figure 2C). However, accuracy dropped considerably when migration was between closely related populations without outgroups present in the data (these are populations 1 and 20 in the model; Figure 2C). The major types of errors produced in the simulations were incorrectly inferred directions of migration arrows and inference of admixture in populations related to the truly admixed population (Text S1, Figure S7).

We next asked whether the mixture “weights” inferred in the model can be interpreted as admixture proportions. To do this, we simulated admixture events of varying proportion between the first and tenth population in the serial bottleneck model described above, set the graph to the true topology, and estimated the mixture weight. The weights are indeed correlated with the true ancestry fraction, but underestimate relatively high admixture proportions in these simulations (Figure 2D). The precise bias in the estimation of this parameter will depend, in real data, on largely unknowable parameters (Text S1).

### Application to humans

**Figure 3:**
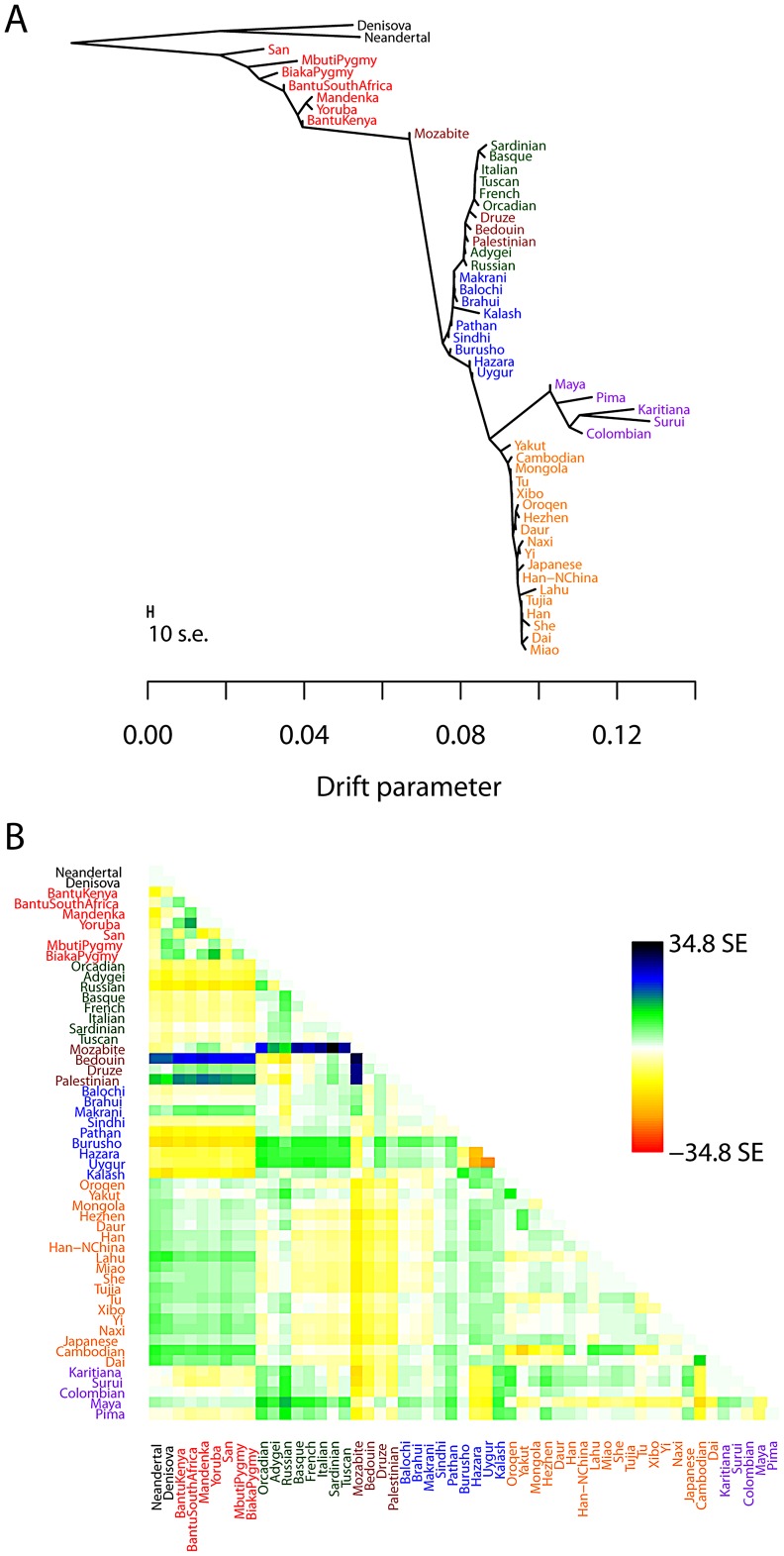
Inferred human tree. A. Maximum likelihood tree. Plotted is the maximum-likelihood tree. Populations are colored according to geographic location (black: archaic humans, red: Africa, brown: Middle East, green: Europe, blue: Central Asia, purple: America, orange: East Asia). The scale bar shows ten times the average standard error of the entries in the sample covariance matrix (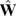). For analysis including Oceania, see Figures S11 and S12. B. Residual fit. Plotted is the residual fit from the maximum likelihood tree in A. We divided the residual covariance between each pair of populations 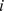 and 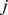 by the average standard error across all pairs. We then plot in each cell 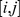 this scaled residual. Colors are described in the palette on the right. Residuals above zero represent populations that are more closely related to each other in the data than in the best-fit tree, and thus are candidates for admixture events.

To test the performance of the *TreeMix* model with real data, we applied it to humans, whose genetic history has been studied extensively [7], [21], [52], [53]. We applied the model to a dataset consisting of about 125,000 SNPs ascertained by low-coverage genome sequencing in a single Yoruban individual and then genotyped in 55 modern and archaic human populations [54]. In all that follows, we excluded the two Oceanian populations because they gave inconsistent results across datasets. We believe this difficulty results from the fact that these populations contain ancestry from multiple sources, making the graph estimation somewhat unstable when they are included (Text S1, Figure S12). We first built the tree of all 53 remaining populations (Figure 3A). This tree largely recapitulates the known relationships among population groups [7], and explains 98.8% of the variance in relatedness between populations (though this is high, it is less than the 99.8% observed in the simulations of a true tree model). We examined the residuals of the model's fit to identify aspects of ancestry not captured by the tree (Figure 3B). A number of known admixed populations stand out: in particular, these include the Mozabite and Middle Eastern populations.

**Figure 4:**
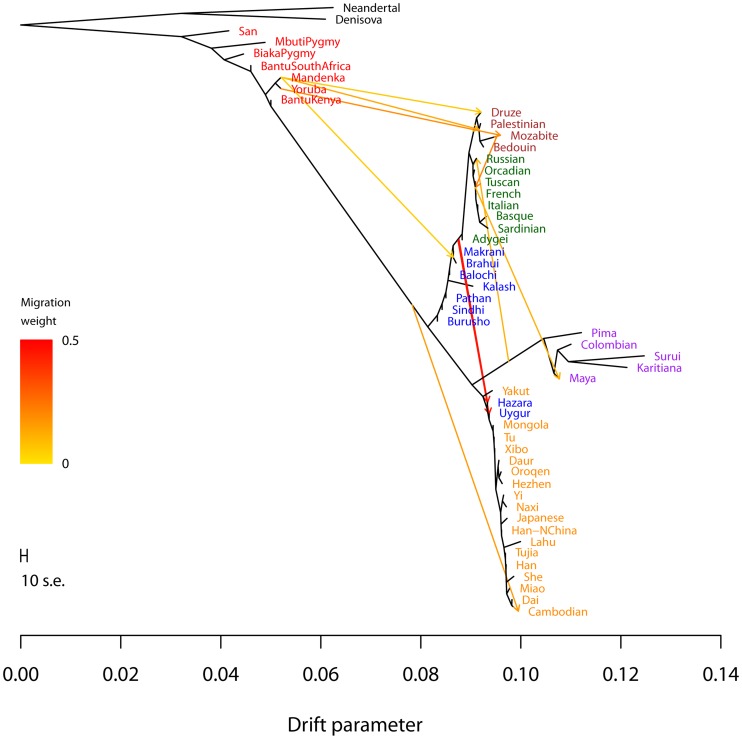
Inferred human tree with mixture events. Plotted is the structure of the graph inferred by *TreeMix* for human populations, allowing ten migration events. Migration arrows are colored according to their weight. Horizontal branch lengths are proportional to the amount of genetic drift that has occurred on the branch. The scale bar shows ten times the average standard error of the entries in the sample covariance matrix (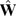). The residual fit from this graph is shown in Figure S9. Admixture from Neandertals to non-African populations is only apparent when considering subsets of the data (see Discussion and Figure S15).

We then sequentially added migration events to the tree. In Figure 4, we show the inferred graph with ten migration edges; p-values for all reported migration edges are less than 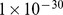 (we show the graph with the maximum likelihood over several independent runs of *TreeMix* with random orders of input populations). This graph model explains 99.8% of the variance in relatedness between populations. As expected from examination of Figure 3B, the migration events recapitulate many known events in human history. We infer that the Mozabite are the result of admixture between an African and a Middle Eastern population (with about 33% of their ancestry from Africa), and that Middle Eastern populations also have African ancestry (Palestinians and Bedouins: 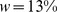 from Africa; Druze: 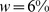). This is consistent with previously reported admixture proportions from these populations [43], [55]. Additionally, we identify the known European ancestry in the Maya (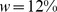) [21], and infer that the Uyghur and Hazara populations are the result of admixture between west Eurasian and East Asian populations (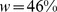 and 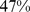 from west Eurasia, respectively) [20], [21], [56].

Several additional migration events in the human data have not been previously examined in detail, but are consistent with previous clustering analysis of these populations [7], [20], [21]. These include migration from Africa to the Makrani and Brahui in Central Asia (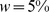) and from a population related to East Asians and Native Americans (which we interpret as likely Siberian) to Russia (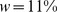).

Two inferred edges were unexpected. First, perhaps the most surprising inference is that Cambodians trace about 16% of their ancestry to a population equally related to both Europeans and other East Asians (while the remaining 84% of their ancestry is related to other southeast Asians). This is partially consistent with clustering analyses, which indicate shared ancestry between Cambodians and central Asian populations [7]. To confirm that the Cambodians are admixed, we turned to less parameterized models. The predicted admixture event implies that allele frequencies in Cambodia are more similar to those in African populations than would be expected based on their East Asian ancestry. To test this, we used three-population tests [37]. We tested the trees [African, [Cambodian,Dai]] for evidence of admixture in the Cambodians (Methods). When using any African population, there is strong evidence of admixture (when using Yoruba, 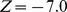 [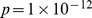]; when using Mandenka, 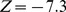 [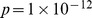]; when using San, 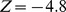 [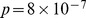]). We conclude that the Cambodian population is the result of an admixture event involving a southeast Asian population related to the Dai and a Eurasian population only distantly related to those present in these data.

Finally, we infer an admixture edge from the Middle East (a population related to the Mozabite, a Berber population from northern Africa) to southern European populations (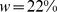). This migration edge is the one edge that is not consistent across independent runs of *TreeMix* on these data (Figure S8). In particular, an alternative graph (albeit with lower likelihood) places the Mozabite as an admixture between southern Europe and Africa (rather than the Middle East and Africa), and does not include an edge from the middle East to southern Europe. We thus hesitate to interpret this result, except to note that the relationship between northern African, the Middle East, and southern Europe involves complex patterns of gene flow that merit further investigation [43], [57].

To test the robustness of our results to SNP ascertainment, we additionally ran *TreeMix* on the same set of populations using a set of SNPs ascertained in a single French individual. The inferred graph was nearly identical (Figure S10). Additionally, as noted above, different random input orders for the populations gave very similar results (Figure S8). We conclude from this that the model is able to consistently and accurately infer the major mixture events in the history of a species. This approach is computationally efficient: building the tree took around five minutes on a standard desktop computer (with a processor speed of 3.1 GHz), and adding ten migration events to the tree took about four and a half hours (the major computational cost is in the iterative estimation of migration weights).

### Application to dogs

**Figure 5:**
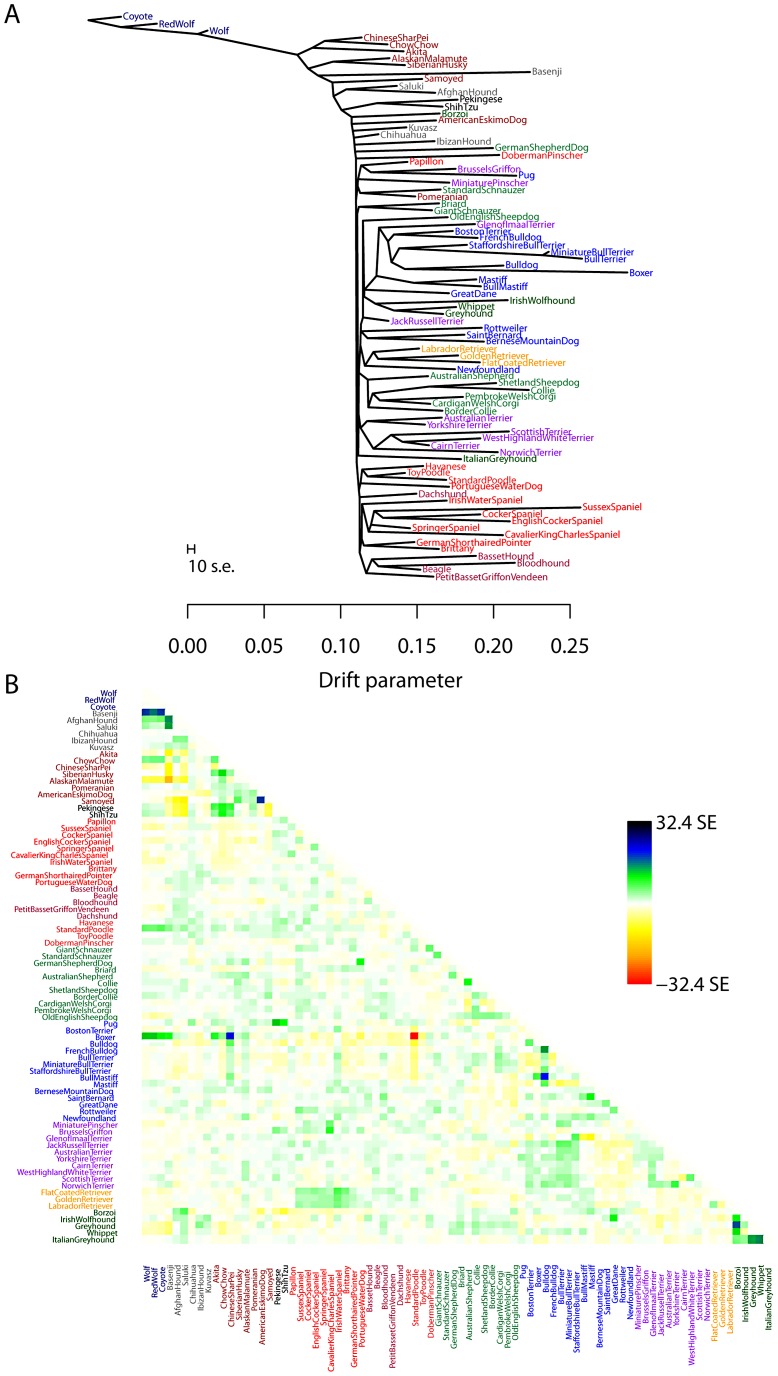
Inferred dog tree. A. Maximum likelihood tree. Populations are colored according to breed type. Dark blue: wild canids, grey: ancient breeds, brown: spitz breeds, black: toy dogs, red: spaniels, maroon: scent hounds, dark red: working dogs, light green: herding dogs, light blue: mastiff-like dogs, purple: small terriers, orange: retrievers, dark green: sight hounds. The scale bar shows ten times the average standard error of the entries in the sample covariance matrix (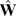). B. Residual fit. Plotted is the residual fit from the maximum likelihood tree in A. We divided the residual covariance between each pair of populations 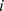 and 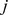 by the average standard error across all pairs. We then plot in each cell 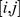 this scaled residual. Colors are described in the palette on the right.

While human populations have been extensively studied, we next applied the model to dogs, a species where considerably less is known about population history. In particular, we applied the model to a dataset consisting of about 60,000 SNPs genotyped in 82 dog breeds or wild canids [58]. As for humans, we first inferred the maximum likelihood tree (Figure 5A). The differences in history between dogs and humans are striking: there are long terminal branches leading to each dog breed in the inferred tree (Figure 5A, recall that the terminal branch lengths account for sample size). This is consistent with the known strong bottlenecks in the establishment of dog breeds [23]. However, examining the residuals from the model revealed a number of populations that do not fit a strict tree model (Figure 5B); indeed, the tree model explained 94.7% of the variance in relatedness between breeds, somewhat less than between human populations.

**Figure 6:**
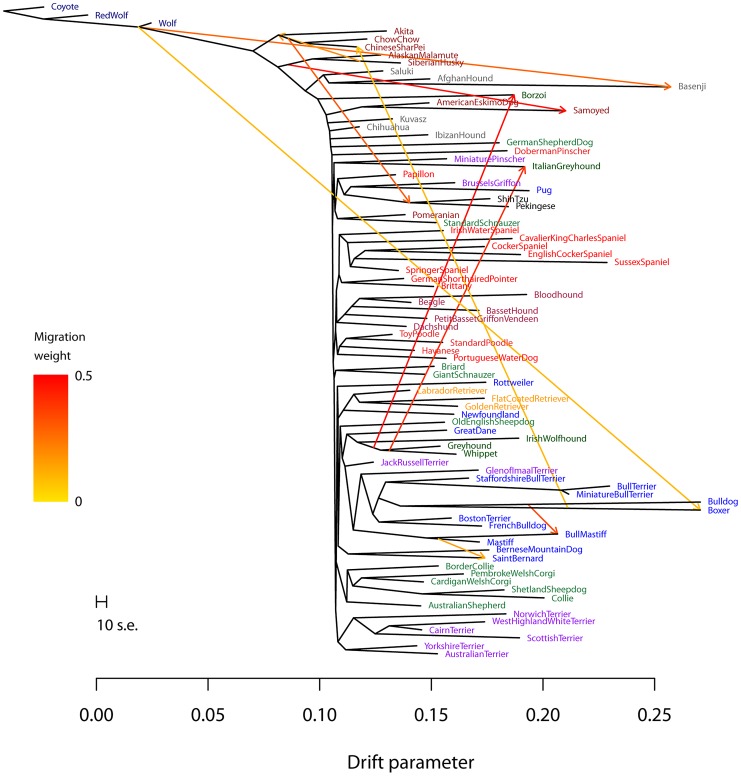
Inferred dog graph. Plotted is the structure of the graph inferred by *TreeMix* for dog populations, allowing ten migration events. Migration arrows are colored according to their weight. The scale bar shows ten times the average standard error of the entries in the sample covariance matrix (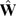). See the main text for discussion. The residual fit from this graph is presented in Figure S13.

We sequentially added migration events to the tree in Figure 5A. In Figure 6, we show the inferred graph with ten migration events, which explains 96.8% of the variance in relatedness between breeds (which suggests that additional events exist in the data). In the following paragraphs, we describe some of these events.

We infer that the bull mastiff is the result of an admixture event between bulldogs and mastiffs. This is a known event [59]; we estimate the admixture proportions as 33% bulldog and 67% mastiff. We further examined this event using four-population tests for treeness. As expected given the known history, the tree [[boxer,bulldog],[mastiff,bull mastiff]] fails the four-population test (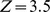, 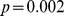), while replacing the bull mastiff with other related breeds that we do not predict to be involved in the admixture event results in trees that pass this test. For example, the tree [[boxer,bulldog],[mastiff,Boston terrier]] passes the four-population test with 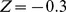.

The most visually apparent residuals in Figure 5B are accounted for in the graph by an admixture event from the grey wolf into the basenji, an ancient African breed of dog (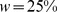). Such a high mixture fraction is consistent with previous clustering analyses of these data [23], [60]. We again sought to confirm this signal in a less-parameterized model. We tested the four-population tree [[wolf,ancient breed],[basenji, Afghan hound]] with various “ancient” dog breeds. We could not find a tree that passed the four-population test (with Akita as the ancient breed, 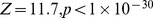; with Alaskan Malamute, 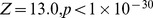), confirming the presence of gene flow in these trees. Replacing the basenji with the saluki in these analyses resulted in trees that pass the four-population test (for example, the tree [[wolf, Akita],[Afghan hound, saluki]] passes with 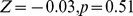). Though we cannot have complete confidence in the precise migration events, these results are consistent with admixture between gray wolves and the basenji.

Another breed that stands out in this analysis is the boxer (Note that many of the SNPs used in this study were ascertained using a boxer individual, so we may have increased power to identify migration events involving this breed). We infer a significant genetic contribution from wolves to the boxer (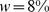), and migration between the boxer and the Chinese shar-pei, a distantly-related ancient breed (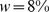). To further examine these events, we again turned to four-population tests. To evaluate the wolf mixture, we tested the tree [[wolf, ancient breed],[boxer, bulldog]]. We did not find a tree that passed the four-population test (with Akita as the ancient breed, 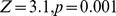; with Afghan Hound, 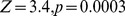). Replacing the Boxer with the Mastiff in these analyses led to trees that passed the four-population test (for example, with Akita as the ancient breed, 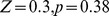). To evaluate the gene flow from the Boxer to the Chinese shar-pei, we tested the tree [[Chinese shar-pei, Akita],[boxer, bulldog]]; this tree fails the four-population test (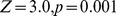), while the tree [[Chow Chow, Akita],[boxer,bulldog]] passes (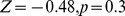).

Previous analyses of these data have noted that the “toy breeds” of dog cluster together Vonholdt:2010uq. We find that the Chinese toy breeds (the Pekingese and the Shi Tzu) result from admixture between a population related to ancient East Asian dog breeds and a modern population related to the Brussels griffon and the pug (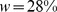 from the East Asian breeds). To confirm the presence of gene flow, we tested four-population trees of the form [[Asian toy breed, Akita/Chow Chow],[Pug,mastiff]]. These trees fail, with varying levels of significance, ranging from [[Chow Chow, Shi Tzu],[Pug, mastiff]] (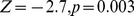) to [[Akita, Pekingese],[Pug, mastiff]] (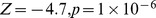).

Finally, we noticed that two of the sighthounds (the Borzoi and the Italian greyhound) do not cluster with the other sight hounds in the tree, namely greyhound, whippet and Irish wolfhound (Figure 5A); however, they do show evidence of having sighthound admixture in the graph (Figure 6). These are the borzoi (which appear to be admixed between an ancient or spitz-breed dog, with 47% ancestry from the sighthounds) and the Italian Greyhound (which appears to be admixed with a toy breed, with 34% ancestry from the sighthounds). This is consistent with the known phenotypic characteristics of these dogs; the borzoi is considered a saluki-like breed, and the Italian greyhound is phenotypically a small version of a greyhound [59].

Overall, we conclude that there has been considerable gene flow between dog breeds over the course of domestication; there are many additional migration events that merit further examination (Figure 6, Text S1).

## Discussion

In this paper, we have developed a unified model for inferring patterns of population splits and mixtures from genome-wide allele frequency data. We have shown that this model is accurate in simulations, largely recapitulates the known relationships between well-studied human populations, and is able to identify new relationships between populations in both humans and dogs.

The *TreeMix* model can be thought of as a complement to methods for the identification of population structure [18]–[20]. These latter methods are powerful tools for clustering together individuals into relatively homogenous populations (and to identify individuals that are genetic outliers in their population) [18]–[20]. However, once population structure in a species has been identified, these methods are not well-suited for describing *how* it arose, and are only indirectly informative about the historical relationships between different populations. The model developed in this paper is designed to more directly address these historical questions.

### Modeling assumptions

There are a number of assumptions, both implicit and explicit, in the interpretation of the *TreeMix* model. First, we have motivated the model in terms of inferring the historical splits and mixtures of populations. However, a given covariance structure of allele frequencies between populations can be a consequence of either a non-equilibrium demography (population splits and mixtures) or an equilibrium demography (populations at long-term stasis with a fixed migration structure) [2]. For the species analyzed in this paper, population equilibrium over the entire species range is not a tenable hypothesis; however, some subsets of populations may be at equilibrium, and there may be species where this alternative historical interpretation of the model is plausible.

We have also modeled migration between populations as occurring at single, instantaneous time points. This is, of course, a dramatic simplification of the migration process. This model will work best when gene flow between populations is restricted to a relatively short time period. Situations of continuous migration violate this assumption and lead to unclear results (Figure S14). The relevance of this assumption will depend on the species and the populations considered. In humans, the relevance of continuous versus discrete mixture events is an open question–some aspects of genetic variation appear compatible with continuous migration [61], while other aspects do not [37]. Indeed, both sorts of models are likely relevant at different time scales [62].

We also rely on the implicit assumption that the history of the species being analyzed is largely tree-like. We have made this assumption to simplify the search for the maximum likelihood graph; additionally, we speculate that in graphs with complex structure, there will be many graphs that lead to identical covariance matrices, and thus several different histories will be compatible with the data. That said, improvements to the search algorithm could allow the assumption of approximate treeness to be somewhat relaxed. Currently, if the number of admixed populations is large relative to the number of unadmixed populations, this assumption breaks down. For example, in the human data, note that we see no evidence of the documented gene flow from Neandertals to all non-African populations [39] (Figure 3B). The reason for this is that the large number of populations with admixture can be accommodated in the tree by allowing the branch from Neandertals to Africans to be slightly underestimated (additionally, by using SNPs ascertained in Africa, we have selected against sites that are informative about Neandertal ancestry). If only a single non-African population is included in the analysis, the relationship between Neandertals and the non-African population is clearer (Figure S15).

### Conclusions

A number of extensions to the sort of model described here are of potential interest. First, the historical relationships between populations could be useful as null demographic models for the detection of natural selection [48], [63], [64]. Second, in a given individual, the best-fit graph relating the individual to other populations may change along a chromosome; this sort of information could be of use in local ancestry inference. Finally, we have not used the information about demographic history present in linkage disequilibrium; approaches that explicitly use this information may provide additional power to detect migration events and estimate their timing, at an additional computational cost [20], [53], [65].

## Methods

### Graph estimation

As described in the Results, we developed an algorithm called *TreeMix* that uses the composite likelihood in Equation 28 to search for the maximum likelihood graph. Estimation involves two major steps. First, for a given graph topology, we need to find the maximum likelihood branch lengths and migration weights. Second, we need to search the space of possible graphs. First consider a given graph topology. We iterate between optimizing the branch lengths and weights. If the edge weights are known, the observed entries of the covariance matrix can be written down as an overdetermined system of linear equations (as in Equations 13–15). We solve this system by non-negative least squares [66]. Though the least squares solution is the maximum composite likelihood solution in the case where all entries of the covariance matrix have equal variance, it is not strictly the maximum likelihood solution in cases with unequal variances. The algorithm could be extended to unequal variances using a weighted least squares approach, but we have not implemented this. We then do a golden section search for the optimal weight (between zero and one) on each migration edge [67]. At each step in the golden section search, we update the branch lengths. We optimize the weight of each migration edge in turn, and iterate over migration edges until convergence.

To search the space of possible graphs, we take a hill-climbing approach. We start by finding a local optimum tree, taking an algorithmic approach similar to Felsenstein [30]. We randomly select three populations, optimize the branch lengths for all three possible trees, and choose the best (in terms of the composite likelihood) tree. Then, we add the remaining populations one by one in a random order. To add a population, we try attaching it to all branches of the current tree, optimizing the branch lengths for each one as described above, and find the most likely spot. We then perform a round of local rearrangements (i.e., nearest-neighbor interchanges [50]) around each internal node, keeping the resulting tree only if it increases the likelihood.

After adding all populations, we calculate the residual covariance matrix, 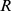. We then add migration edges in a directed matter. First, we find the 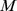 pairs of populations with the maximum residuals (these are the pairs of populations with the worst fit under the model). In the results reported, 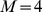. We define a “neighborhood” around each population of a pair as the tips within a distance of 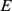 edges of the focal population. In applications above, we use 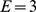. This defines a set of pairs of populations that either have a poor fit, or are located in the graph near populations with a poor fit. We take each of these pairs in turn. For each pair, we identify the set of nodes in the path from each member of the pair to the root of the graph. This gives us two sets of nodes. We take all pairwise combinations of nodes in each set, and look at residuals between the populations that are the descendants of each node. If all of the residuals are positive, we add a migration edge between the two nodes and estimate its maximum likelihood weight. We then keep only the single edge that most increases the likelihood of the graph. After adding a migration edge, we attempt nearest-neighbor interchanges at the source and destination of the migration event, attempt changing the source and destination of all migration events, and attempt changing the direction of all migration arrows. Once we have reached the local maximum by this method, we attempt nearest-neighbor interchanges at all internal nodes. We iterate over this procedure for a predetermined number of migration edges. We then test the migration edges for significance as described.

The *TreeMix* source code is available at http://treemix.googlecode.com.

### Three- and four-population tests of treeness

We implemented three- and four-population tests as described in Reich et al. [37]. For the relationship between the 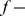 statistics and the covariance model underlying *TreeMix*, see the Text S1. For the three-population test, we estimated 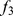 as in Reich et al. [37], and tested whether is it less than zero. We report the Z-score for this test. To obtain a standard error on the estimate of 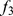, we used a block jackknife similar to Reich et al. [37]. However, Reich et al. [37] split the genome into blocks based on distance (with variable numbers of SNPs per block); we split the genome into blocks of 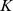 SNPs (and thus the blocks will be of variable size).

For the four-population test for treeness, we calculate the 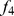 statistic as in Reich et al. [37], and test whether it is different than zero. Again, we report a Z-score for this test. Standard errors for the 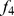 statistic were obtained as for the 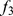 statistic.

### Human data

The human data we used were downloaded from http://www.cephb.fr/en/hgdp/ on August 16th, 2011 (the data set labeled Harvard HGDP-CEPH genotypes). They consist of several panels of SNPs ascertained from low-coverage genome sequencing of single individual from different populations and then genotyped in the Human Genome Diversity Panel [54]. Additionally, at each site, a single sequencing read from the Denisova and Neandertal genome sequencing projects was sampled and the allele reported. These data have the property that they allow for complete control of the ascertainment strategy, and allow us to test the robustness of inference to different ascertainment schemes. For the main analyses, we used the panel of autosomal SNPs ascertained in a single Yoruban individual; there are 124,115 such sites. For some analyses, we also used the panel of autosomal SNPs ascertained in a single French individual; there are 111,970 such sites. For all analyses with *TreeMix*, we used a window size (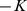) of 500; this corresponds to a window size of approximately 10 Mb. For all *TreeMix* analyses, we set the Neandertal and Denisova samples as the outgroups.

Since we have only a single allele from the Neandertal and Denisova populations, we cannot calculate heterozygosity in these populations for unbiased estimation of the covariance matrix (see ). To account for this, we simply chose a relatively low level of heterozygosity and assigned it to both populations. In the Yoruba ascertained SNPs, we used a heterozygosity of 0.13, and for the French ascertained SNPs, we used a heterozygosity of 0.2. In practice, this only affected the lengths of the terminal branches to Neandertal and Denisova; running *TreeMix* with a heterozygosity of zero in both populations resulted in the same graph topologies (not shown).

### Dog data

Allele counts for the dog breeds and wild canids reported in Boyko et al. Boyko:2010fk were downloaded from http://genome-mirror.bscb.cornell.edu/ on July 30, 2011. These data consist of counts of reference and alternate alleles at 61,468 sites in 85 dog breeds and wild canids. We removed the Jackal and Scottish Deerhound for having relatively high amounts of missing data, and the village dogs because it is unclear if they represent a coherent population. We also removed all SNPs on the X chromosome. This left us with 60,615 SNPs in 82 populations. We ran *TreeMix* with a window size (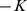) of 500. This corresponds to a window size of approximately 20 Mb. For all *TreeMix* analyses, we set the coyote as the outgroup.

The ascertainment scheme used for SNP discovery in dogs was complicated [68]. The largest set of SNPs were ascertained by virtue of being different between the boxer and poodle assemblies. This should lead to an overestimation of the distance between the boxer and the poodle in our analysis. Indeed, in Figure 5B, a considerable negative residual between the boxer and poodle is visible. Another set of SNPs were ascertained by being heterozygous within a boxer individual, and a third set were ascertained by comparison between a boxer and wild canids. These latter SNPs should lead to an overestimation of the distance between the boxer and the wolf in our analysis (as we see for the poodle); in fact, we infer migration between the boxer and the wolf. This ascertainment issue may have led us to underestimate the amount of gene flow in the comparison.

### Simulations

All simulations were performed using *ms* [69]. The exact commands used are listed in Text S1. When running *TreeMix* on simulations without ascertainment, we used a window size of 5000 SNPs; for simulations with ascertainment we used windows of 1000 SNPs. Consensus trees were generated using SumTrees v.3.1.0 [70].

## Supporting Information

Figure S1 A graph with a mixture event. Capital letters represent nodes, branch length parameters are in blue, and weight parameters are in red.
(PDF)

Figure S2 Replicates of inferred trees from simulated data. We generated tree-like data using the topology in Figure 2A in the main text. In Figure 2B in the main text, we show the inferred tree with mean branch lengths. In this figure, we show four representative individual trees.
(PDF)

Figure S3 Inferred trees on ascertained data. We generated tree-like data using the topology in Figure 2A in the main text. We then used only the SNPs that were polymorphic in either population 1 (A.) or population 20 (B.) to infer the trees. The correct topology was obtained in all 100 simulations; the branch lengths in each figure are the mean across all simulations.
(PDF)

Figure S4 Histogram of p-values for migration in simulated data. We generated 100 tree-like datasets using the topology in Figure 2A in the main text. We then randomly chose two populations (without replacement), added a migration edge between the two populations, and tested for significance using the procedure described in the main text. Plotted is the histogram of p-values for the significance test. If the p-values are properly calibrated, this distribution should be uniform (dotted line). Though the distribution is not completely uniform, there is no skew towards low p-values.
(PDF)

Figure S5 Consensus tree in simulations with long branches. We generated 100 tree-like datasets using the topology in Figure 2A in the main text, multiplying all branch lengths by 50. We then inferred the maximum likelihood tree. A. Plotted are the mean branch lengths from the simulations. All simulations resulted in the same inferred topology. B. In each simulation, we scaled the residuals by the average standard error, then averaged these scaled residuals across simulations. Plotted are the mean scaled residuals across the 100 simulations. The most extreme residuals are not large (around 0.3 standard errors), but tend to be present between closely related populations.
(PDF)

Figure S6 Example trees from simulations with long branches. We generated 100 tree-like datasets using the topology in Figure 2A in the main text, multiplying all branch lengths by 50. We then inferred the maximum likelihood tree. In Figure S5, we show the average inferred tree. Here, we show two representative trees (A. and C. and the residuals corresponding to each tree (B. and D.).
(PDF)

Figure S7 Representative errors in simulations. We examined the simulations in which *TreeMix* did not reach the correct answer. A. The correct topology for the simulations presented in the other panels. B. A representative example of an incorrect topology inferred from the simulations of a migration event with weight 10% from population 1 to population 5 (this topology accounted for all observed errors). C. A representative example of an incorrect topology inferred from the simulations of a migration event with weight 30% from population 1 to population 5 (this topology accounted for 95% of all errors).
(PDF)

Figure S8 Replicate graphs inferred in the human data. These graphs were generated in an identical manner as Figure 4 in the main text, but using different random input orders for populations during tree-building. All random input orders gave very similar results.
(PDF)

Figure S9 Residual fit from graph of human data presented in the main text. Plotted are the residuals from the fit of the graph presented in Figure 4 in the main text.
(PDF)

Figure S10 Graph inferred from SNPs ascertained in a single French individual. The graph was generated in an identical manner as Figure 4 in the main text, but using a panel of SNPs ascertained in a single French, rather than a single Yoruban, individual. The inferred graph is extremely similar to that in Figure 4. The one major difference is that, in this graph, the Mozabite appear as an admixture of a Sardinian population rather than a Middle Eastern population; this configuration is seen in some runs of *TreeMix* on the Yoruba-ascertained data (Figure S8A).
(PDF)

Figure S11 Trees inferred using the human data including the Oceanians. We show the maximum likelihood trees and residuals for the human data including the Oceanian populations, plotted in the same manner as in Figure 3 in the main text. Trees were inferred using the panel of SNPs ascertained in a single Yoruban individual (A. and C.) and the panel of SNPs ascertained in a single French individual (B. and D.).
(PDF)

Figure S12 Graphs inferred using the human data including the Oceanians. We show the maximum likelihood graphs for the human data including the Oceanian populations, plotted in the same manner as in Figure 4 in the main text. Six migration edges were inferred in each graph. Graphs were inferred using the panel of SNPs ascertained in a single Yoruban individual (A.) and the panel of SNPs ascertained in a single French individual (B.). See Text S1 for discussion.
(PDF)

Figure S13 Residual fit from graph of dog data presented in the main text. Plotted are the residuals from the fit of the graph presented in Figure 6 in the main text.
(PDF)

Figure S14
*TreeMix* run on populations with continuous migration. We simulated a set of populations on a lattice, where each population has constant gene flow at a rate of 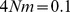 with neighboring populations. All populations split from an outgroup 16,400 generations in the past. The exact *ms* command is given in Text S1. The configuration of the lattice is presented in A. (population 1 is the outgroup). *TreeMix* inferred no consistent tree structure. Three representative trees are presented in B.-D.(PDF)

Figure S15
*TreeMix* run on human data using only a single non-African population. We inferred the maximum likelihood tree (A.) using only the African populations and one non-African population (French), using SNPs identified in a single Yoruban individual. In examining the residuals (B.), a relationship between the French and the Neandertal is clear. We then inferred three migration events (C.), where we do see that the French contain some Neandertal ancestry (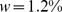). Residual fit for this graph is shown in D.(PDF)

Text S1 Supplementary Information.
(PDF)

## References

[1] Cavalli-Sforza LL, Edwards AW (1967) Phylogenetic analysis. Models and estimation proce-dures. Am J Hum Genet 19: 233–57. 6026583 PMC1706274

[2] Felsenstein J (1982) How can we infer geography and history from gene frequencies? J Theor Biol 96: 9–20. 7109659 10.1016/0022-5193(82)90152-7

[3] Cann RL, Stoneking M, Wilson AC (1987) Mitochondrial DNA and human evolution. Nature 325: 31–6. 3025745 10.1038/325031a0

[4] Nei M, Roychoudhury AK (1974) Genic variation within and between the three major races of man, Caucasoids, Negroids, and Mongoloids. Am J Hum Genet 26: 421–43. 4841634 PMC1762596

[5] Nei M, Roychoudhury AK (1993) Evolutionary relationships of human populations on a global scale. Mol Biol Evol 10: 927–43. 8412653 10.1093/oxfordjournals.molbev.a040059

[6] Cavalli-Sforza LL, Piazza A, Menozzi P, Mountain J (1988) Reconstruction of human evolution: bringing together genetic, archaeological, and linguistic data. Proc Natl Acad Sci U S A 85: 6002–6. 3166138 10.1073/pnas.85.16.6002 PMC281893

[7] Li JZ, Absher DM, Tang H, Southwick AM, Casto AM, et al (2008) Worldwide human rela-tionships inferred from genome-wide patterns of variation. Science 319: 1100–1104. 18292342 10.1126/science.1153717

[8] Pritchard JK, Seielstad MT, Perez-Lezaun A, Feldman MW (1999) Population growth of human Y chromosomes: a study of Y chromosome microsatellites. Mol Biol Evol 16: 1791–8. 10605120 10.1093/oxfordjournals.molbev.a026091

[9] Beaumont MA, Zhang W, Balding DJ (2002) Approximate Bayesian computation in popula-tion genetics. Genetics 162: 2025–35. 12524368 10.1093/genetics/162.4.2025 PMC1462356

[10] Schaffner SF, Foo C, Gabriel S, Reich D, Daly MJ, et al (2005) Calibrating a coalescent simulation of human genome sequence variation. Genome Res 15: 1576–1583. 16251467 10.1101/gr.3709305 PMC1310645

[11] Gutenkunst RN, Hernandez RD, Williamson SH, Bustamante CD (2009) Inferring the joint demographic history of multiple populations from multidimensional SNP frequency data. PLoS Genet 5: e1000695 doi:10.1371/journal.pgen.1000695. 19851460 10.1371/journal.pgen.1000695 PMC2760211

[12] Hey J, Nielsen R (2004) Multilocus methods for estimating population sizes, migration rates and divergence time, with applications to the divergence of Drosophila pseudoobscura and D. persimilis. Genetics 167: 747–60. 15238526 10.1534/genetics.103.024182 PMC1470901

[13] Beerli P, Felsenstein J (1999) Maximum-likelihood estimation of migration rates and effective population numbers in two populations using a coalescent approach. Genetics 152: 763–73. 10353916 10.1093/genetics/152.2.763 PMC1460627

[14] Becquet C, Przeworski M (2007) A new approach to estimate parameters of speciation models with application to apes. Genome Res 17: 1505–19. 17712021 10.1101/gr.6409707 PMC1987350

[15] Kubatko LS (2009) Identifying hybridization events in the presence of coalescence via model selection. Syst Biol 58: 478–88. 20525602 10.1093/sysbio/syp055

[16] Gronau I, Hubisz MJ, Gulko B, Danko CG, Siepel A (2011) Bayesian inference of ancient human demography from individual genome sequences. Nat Genet 43: 1031–4. 21926973 10.1038/ng.937 PMC3245873

[17] Menozzi P, Piazza A, Cavalli-Sforza L (1978) Synthetic maps of human gene frequencies in Europeans. Science 201: 786–92. 356262 10.1126/science.356262

[18] Pritchard JK, Stephens M, Donnelly P (2000) Inference of population structure using multi-locus genotype data. Genetics 155: 945–59. 10835412 10.1093/genetics/155.2.945 PMC1461096

[19] Patterson N, Price AL, Reich D (2006) Population structure and eigenanalysis. PLoS Genet 2: e190 doi:10.1371/journal.pgen.0020190. 17194218 10.1371/journal.pgen.0020190 PMC1713260

[20] Lawson DJ, Hellenthal G, Myers S, Falush D (2012) Inference of Population Structure using Dense Haplotype Data. PLoS Genet 8: e1002453 doi:10.1371/journal.pgen.1002453. 22291602 10.1371/journal.pgen.1002453 PMC3266881

[21] Rosenberg NA, Pritchard JK, Weber JL, Cann HM, Kidd KK, et al (2002) Genetic structure of human populations. Science 298: 2381–2385. 12493913 10.1126/science.1078311

[22] Liti G, Carter DM, Moses AM, Warringer J, Parts L, et al (2009) Population genomics of domestic and wild yeasts. Nature 458: 337–41. 19212322 10.1038/nature07743 PMC2659681

[23] vonHoldt BM, Pollinger JP, Lohmueller KE, Han E, Parker HG, et al (2010) Genome-wide SNP and haplotype analyses reveal a rich history underlying dog domestication. Nature 464: 898–902. 20237475 10.1038/nature08837 PMC3494089

[24] François O, Currat M, Ray N, Han E, Excoffier L, et al (2010) Principal component analysis under population genetic models of range expansion and admixture. Mol Biol Evol 27: 1257–68. 20097660 10.1093/molbev/msq010

[25] McVean G (2009) A genealogical interpretation of principal components analysis. PLoS Genet 5: e1000686 doi:10.1371/journal.pgen.1000686. 19834557 10.1371/journal.pgen.1000686 PMC2757795

[26] Novembre J, Stephens M (2008) Interpreting principal component analyses of spatial popula-tion genetic variation. Nat Genet 40: 646–9. 18425127 10.1038/ng.139 PMC3989108

[27] Saitou N, Nei M (1987) The neighbor-joining method: a new method for reconstructing phy-logenetic trees. Mol Biol Evol 4: 406–25. 3447015 10.1093/oxfordjournals.molbev.a040454

[28] Felsenstein J (1973) Maximum-likelihood estimation of evolutionary trees from continuous characters. Am J Hum Genet 25: 471–92. 4741844 PMC1762641

[29] RoyChoudhury A, Felsenstein J, Thompson EA (2008) A two-stage pruning algorithm for likelihood computation for a population tree. Genetics 180: 1095–105. 18780754 10.1534/genetics.107.085753 PMC2567359

[30] Felsenstein J (1981) Evolutionary trees from gene frequencies and quantitative characters: finding maximum likelihood estimates. Evolution 35: 1229–1242. 10.1111/j.1558-5646.1981.tb04991.x 28563384

[31] Sirén J, Marttinen P, Corander J (2011) Reconstructing population histories from single nu-cleotide polymorphism data. Mol Biol Evol 28: 673–83. 20819907 10.1093/molbev/msq236

[32] Nielsen R, Mountain JL, Huelsenbeck JP, Slatkin M (1998) Maximum-likelihood estimation of population divergence times and population phylogeny in models without mutation. Evolution 52: 669–677. 10.1111/j.1558-5646.1998.tb03692.x 28565245

[33] Nicholson G, Smith AV, Jónsson F, Gústafsson Ó, Stefánsson K, et al (2002) Assessing Pop-ulation Differentiation and Isolation from Single-Nucleotide Polymorphism Data. Journal of the Royal Statistical Society Series B (Statistical Methodology) 64: 695–715.

[34] Cavalli-Sforza LL (1973) Analytic review: some current problems of human population genet-ics. Am J Hum Genet 25: 82–104. 4567614 PMC1762215

[35] Cavalli-Sforza LL, Piazza A (1975) Analysis of evolution: evolutionary rates, independence and treeness. Theor Popul Biol 8: 127–65. 1198349 10.1016/0040-5809(75)90029-5

[36] Keinan A, Mullikin JC, Patterson N, Reich D (2007) Measurement of the human allele fre-quency spectrum demonstrates greater genetic drift in East Asians than in Europeans. Nat Genet 39: 1251–5. 17828266 10.1038/ng2116 PMC3586588

[37] Reich D, Thangaraj K, Patterson N, Price AL, Singh L (2009) Reconstructing Indian popula-tion history. Nature 461: 489–94. 19779445 10.1038/nature08365 PMC2842210

[38] Durand EY, Patterson N, Reich D, Slatkin M (2011) Testing for Ancient Admixture between Closely Related Populations. Mol Biol Evol 28: 2239–52. 21325092 10.1093/molbev/msr048 PMC3144383

[39] Green RE, Krause J, Briggs AW, Maricic T, Stenzel U, et al (2010) A draft sequence of the Neandertal genome. Science 328: 710–22. 20448178 10.1126/science.1188021 PMC5100745

[40] Lathrop GM (1982) Evolutionary trees and admixture: phylogenetic inference when some populations are hybridized. Ann Hum Genet 46: 245–55. 7125596 10.1111/j.1469-1809.1982.tb00716.x

[41] Reich D, Green RE, Kircher M, Krause J, Patterson N, et al (2010) Genetic history of an archaic hominin group from Denisova Cave in Siberia. Nature 468: 1053–60. 21179161 10.1038/nature09710 PMC4306417

[42] Reich D, Patterson N, Kircher M, Delfin F, Nandineni MR, et al (2011) Denisova admixture and the first modern human dispersals into Southeast Asia and Oceania. Am J Hum Genet 89: 516–28. 21944045 10.1016/j.ajhg.2011.09.005 PMC3188841

[43] Moorjani P, Patterson N, Hirschhorn JN, Keinan A, Hao L, et al (2011) The history of African gene flow into Southern Europeans, Levantines, and Jews. PLoS Genet 7: e1001373 doi:10.1371/journal.pgen.1001373.. 21533020 10.1371/journal.pgen.1001373 PMC3080861

[44] Rasmussen M, Guo X, Wang Y, Lohmueller KE, Rasmussen S, et al (2011) An Aboriginal Australian genome reveals separate human dispersals into Asia. Science 334: 94–8. 21940856 10.1126/science.1211177 PMC3991479

[45] Huson D, Rupp R, Scornavacca C (2010) Phylogenetic Networks. Concepts, Algorithms and Applications. Cambridge University Press.

[46] Huson DH, Bryant D (2006) Application of phylogenetic networks in evolutionary studies. Mol Biol Evol 23: 254–67. 16221896 10.1093/molbev/msj030

[47] Dyer RJ, Nason JD (2004) Population Graphs: the graph theoretic shape of genetic structure. Mol Ecol 13: 1713–27. 15189198 10.1111/j.1365-294X.2004.02177.x

[48] Coop G, Witonsky D, Di Rienzo A, Pritchard JK (2010) Using environmental correlations to identify loci underlying local adaptation. Genetics 185: 1411–23. 20516501 10.1534/genetics.110.114819 PMC2927766

[49] Weir BS, Hill WG (2002) Estimating F-statistics. Annu Rev Genet 36: 721–50. 12359738 10.1146/annurev.genet.36.050802.093940

[50] Felsenstein J (2003) Inferring Phylogenies. Sinauer Associates, 2nd edition.

[51] DeGiorgio M, Jakobsson M, Rosenberg NA (2009) Out of Africa: modern humanorigins special feature: explaining worldwide patterns of human genetic variation using a coalescent- based serial founder model of migration outward from Africa. Proc Natl Acad Sci U S A 106: 16057–62. 19706453 10.1073/pnas.0903341106 PMC2752555

[52] Jakobsson M, Scholz SW, Scheet P, Gibbs JR, VanLiere JM, et al (2008) Genotype, haplotype and copy-number variation in worldwide human populations. Nature 451: 998–1003. 18288195 10.1038/nature06742

[53] Hellenthal G, Auton A, Falush D (2008) Inferring human colonization history using a copying model. PLoS Genet 4: e1000078 doi:10.1371/journal.pgen.1000078.. 18497854 10.1371/journal.pgen.1000078 PMC2367454

[54] Patterson N, Moorjani P, Luo Y, Mallick S, Rohland N, et al (2012) Ancient admixture. Genetics In Press. 10.1534/genetics.112.145037 PMC3522152 22960212

[55] Price AL, Tandon A, Patterson N, Barnes KC, Rafaels N, et al (2009) Sensitive detection of chromosomal segments of distinct ancestry in admixed populations. PLoS Genet 5: e1000519 doi:10.1371/journal.pgen.1000519.. 19543370 10.1371/journal.pgen.1000519 PMC2689842

[56] Xu S, Huang W, Qian J, Jin L (2008) Analysis of genomic admixture in Uyghur and its implication in mapping strategy. Am J Hum Genet 82: 883–94. 18355773 10.1016/j.ajhg.2008.01.017 PMC2427216

[57] Henn BM, Botigué LR, Gravel S, Wang W, Brisbin A, et al (2012) Genomic ancestry of North Africans supports back-to-Africa migrations. PLoS Genet 8: e1002397 doi:10.1371/journal.pgen.1002397.. 22253600 10.1371/journal.pgen.1002397 PMC3257290

[58] Boyko AR, Quignon P, Li L, Schoenebeck JJ, Degenhardt JD, et al (2010) A simple genetic architecture underlies morphological variation in dogs. PLoS Biol 8: e1000451 doi:10.1371/journal.pbio.1000451.. 20711490 10.1371/journal.pbio.1000451 PMC2919785

[59] American Kennel Club (2006) The complete dog book. Ballantine Books.

[60] Parker HG, Kim LV, Sutter NB, Carlson S, Lorentzen TD, et al (2004) Genetic structure of the purebred domestic dog. Science 304: 1160–4. 15155949 10.1126/science.1097406

[61] Novembre J, Johnson T, Bryc K, Kutalik Z, Boyko AR, et al (2008) Genes mirror geography within Europe. Nature 456: 98–101. 18758442 10.1038/nature07331 PMC2735096

[62] Novembre J, Ramachandran S (2011) Perspectives on human population structure at the cusp of the sequencing era. Annu Rev Genomics Hum Genet 12: 245–74. 21801023 10.1146/annurev-genom-090810-183123

[63] Bhatia G, Patterson N, Pasaniuc B, Zaitlen N, Genovese G, et al (2011) Genome-wide com-parison of African-ancestry populations from CARe and other cohorts reveals signals of natural selection. Am J Hum Genet 89: 368–81. 21907010 10.1016/j.ajhg.2011.07.025 PMC3169818

[64] Bonhomme M, Chevalet C, Servin B, Boitard S, Abdallah J, et al (2010) Detecting selection in population trees: the Lewontin and Krakauer test extended. Genetics 186: 241–62. 20855576 10.1534/genetics.110.117275 PMC2940290

[65] Myers S, Hellenthal G, Lawson D, Busby G, Leslie S, et al. (2011). LD patterns in dense variation data reveal information about the history of human populations worldwide. URL http://www.ichg2011.org/cgi-bin/ichg11s?&sort=ptimes&sbutton=Detail&absno=21708&sid=806980.

[66] Lawson CL, Hanson RJ (1995) Solving least squares problems. Philadelphia, PA: Society for Industrial Mathematics, 3rd edition.

[67] Press WH, Teukolsky SA, Vetterling WT, Flannery BP (1992) Numerical recipes in C (2^nd^ ed.): the art of scientific computing. New York, NY, USA: Cambridge University Press.

[68] Lindblad-Toh K, Wade CM, Mikkelsen TS, Karlsson EK, Jaffe DB, et al (2005) Genome sequence, comparative analysis and haplotype structure of the domestic dog. Nature 438: 803–19. 16341006 10.1038/nature04338

[69] Hudson RR (2002) Generating samples under a Wright-Fisher neutral model of genetic varia-tion. Bioinformatics 18: 337–8. 11847089 10.1093/bioinformatics/18.2.337

[70] Sukumaran J, Holder MT (2010) DendroPy: a Python library for phylogenetic computing. Bioinformatics 26: 1569–71. 20421198 10.1093/bioinformatics/btq228

