# Supplementary figures and images for: Inference of Population Splits and Mixtures from Genome-Wide Allele Frequency Data
Source: PLoS Genet. 2012 Nov 15;8(11):e1002967. doi: 10.1371/journal.pgen.1002967 (PMC3499260; doi:10.1371/journal.pgen.1002967)

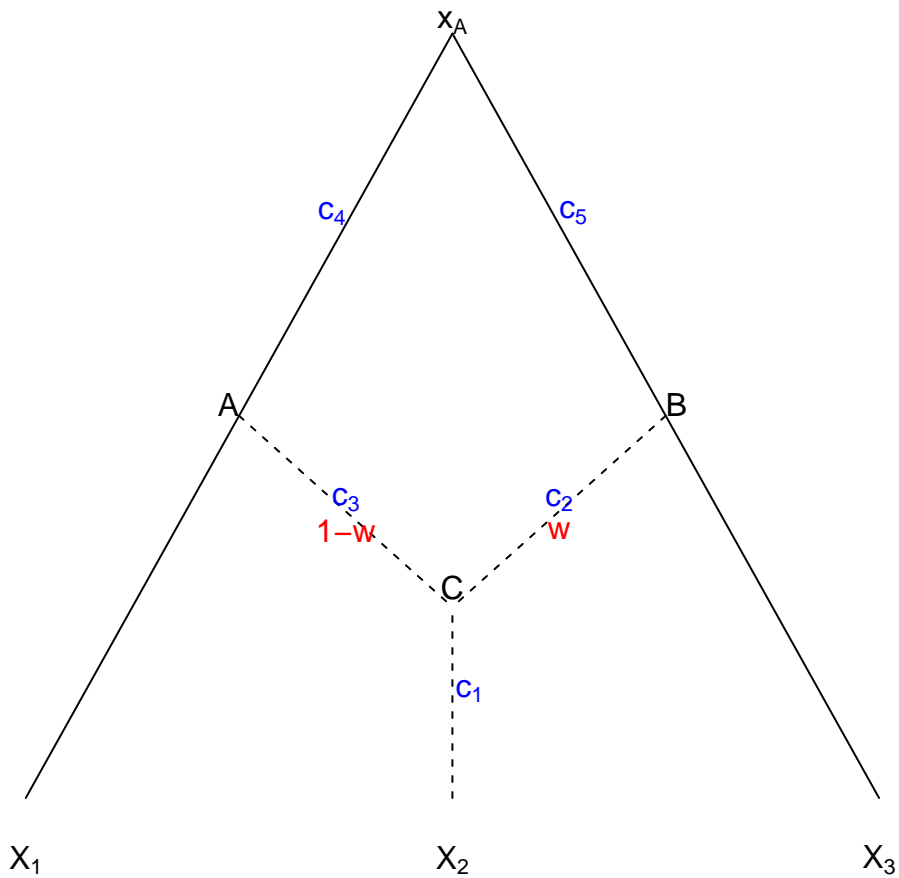

Supplement: Figure S1 — A graph with a mixture event. Capital letters represent nodes, branch length parameters are in blue, and weight parameters are in red. (PDF) [file pgen.1002967.s001.pdf]

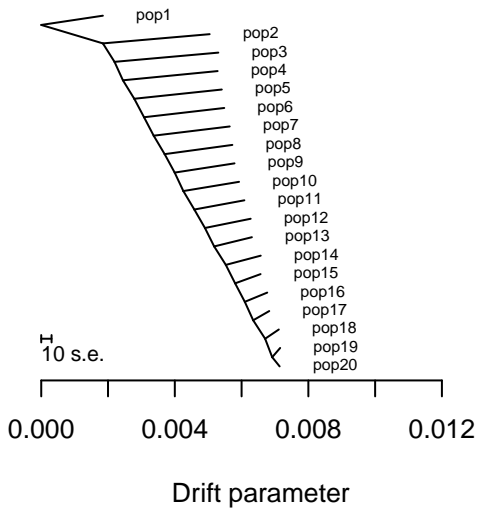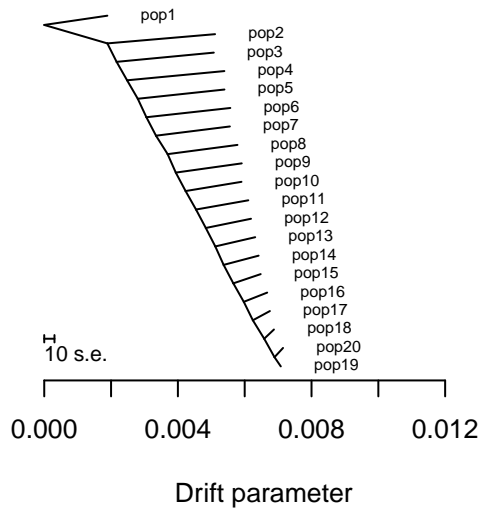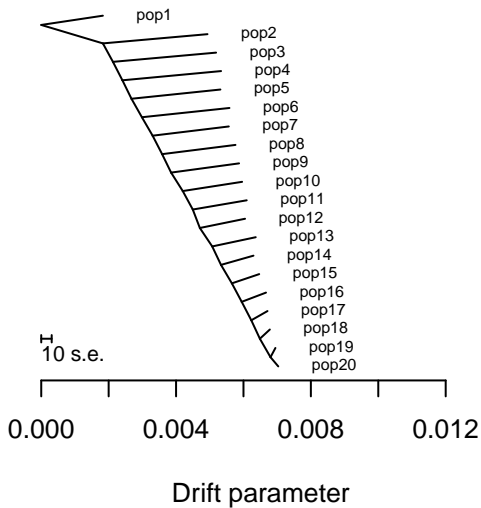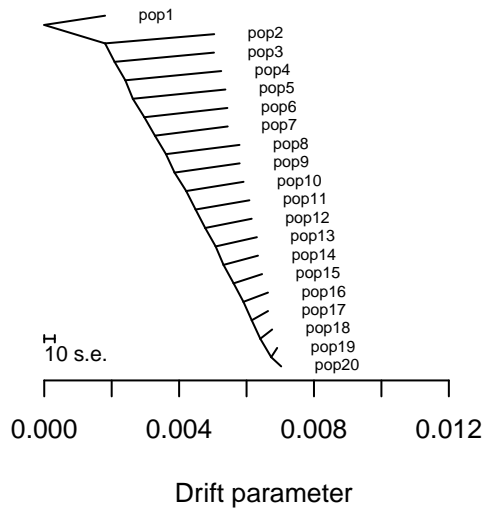

Supplement: Figure S2 — Replicates of inferred trees from simulated data. We generated tree-like data using the topology in Figure 2A in the main text. In Figure 2B in the main text, we show the inferred tree with mean branch lengths. In this figure, we show four representative individual trees. (PDF) [file pgen.1002967.s002.pdf]

A. Ascertainment in pop1

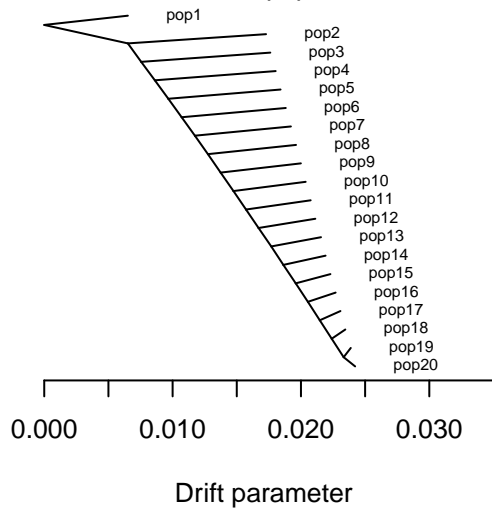

B. Ascertainment in pop20

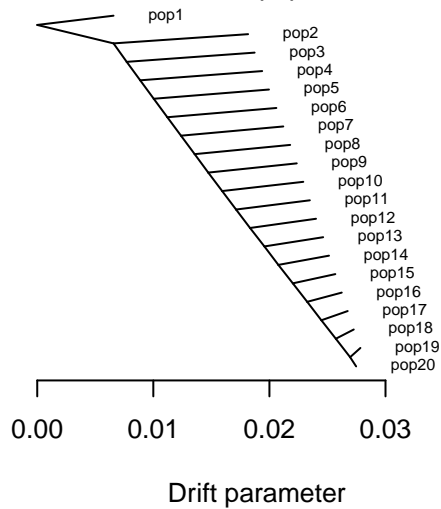

Supplement: Figure S3 — Inferred trees on ascertained data. We generated tree-like data using the topology in Figure 2A in the main text. We then used only the SNPs that were polymorphic in either population 1 (A.) or population 20 (B.) to infer the trees. The correct topology was obtained in all 100 simulations; the branch lengths in each figure are the mean across all simulations. (PDF) [file pgen.1002967.s003.pdf]

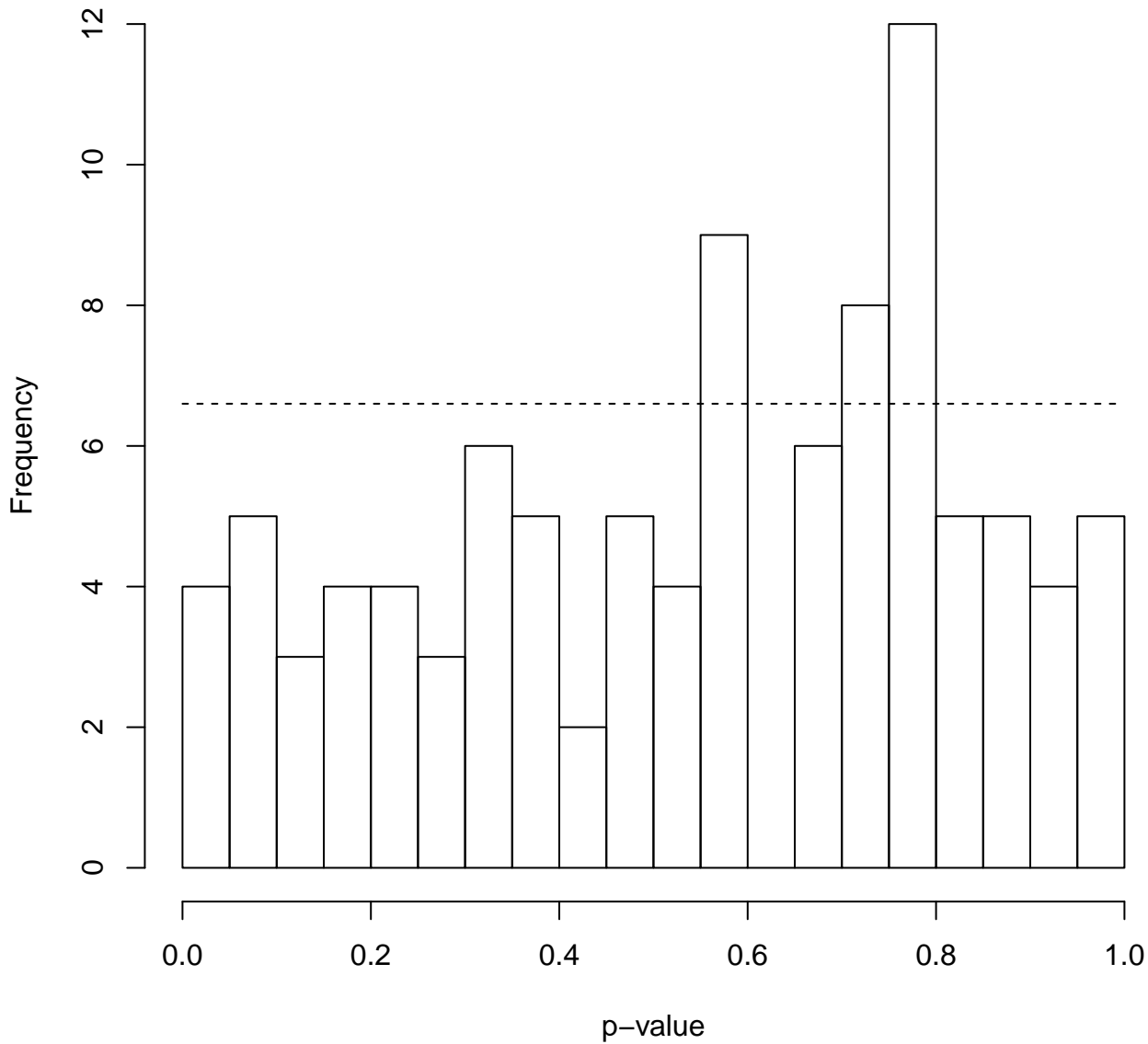

Supplement: Figure S4 — Histogram of p-values for migration in simulated data. We generated 100 tree-like datasets using the topology in Figure 2A in the main text. We then randomly chose two populations (without replacement), added a migration edge between the two populations, and tested for significance using the procedure described in the main text. Plotted is the histogram of p-values for the significance test. If the p-values are properly calibrated, this distribution should be uniform (dotted line). Though the distribution is not completely uniform, there is no skew towards low p-values. (PDF) [file pgen.1002967.s004.pdf]

A. Consensus tree

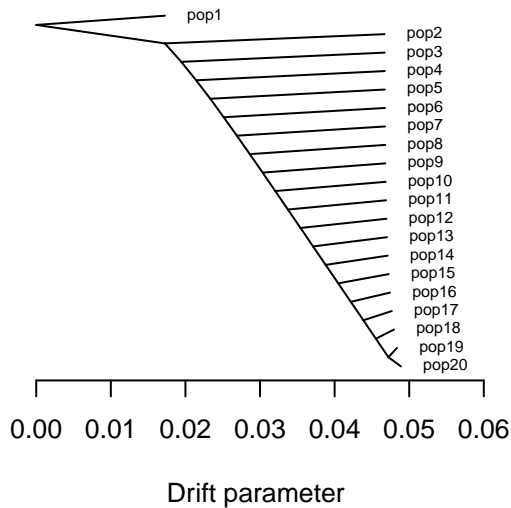

B. Average residuals

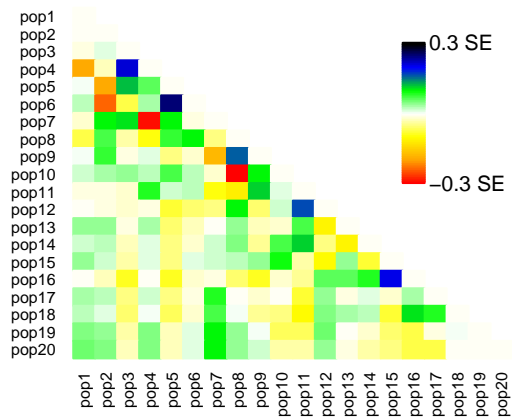

Supplement: Figure S5 — Consensus tree in simulations with long branches. We generated 100 tree-like datasets using the topology in Figure 2A in the main text, multiplying all branch lengths by 50. We then inferred the maximum likelihood tree. A. Plotted are the mean branch lengths from the simulations. All simulations resulted in the same inferred topology. B. In each simulation, we scaled the residuals by the average standard error, then averaged these scaled residuals across simulations. Plotted are the mean scaled residuals across the 100 simulations. The most extreme residuals are not large (around 0.3 standard errors), but tend to be present between closely related populations. (PDF) [file pgen.1002967.s005.pdf]

A. Example tree 1

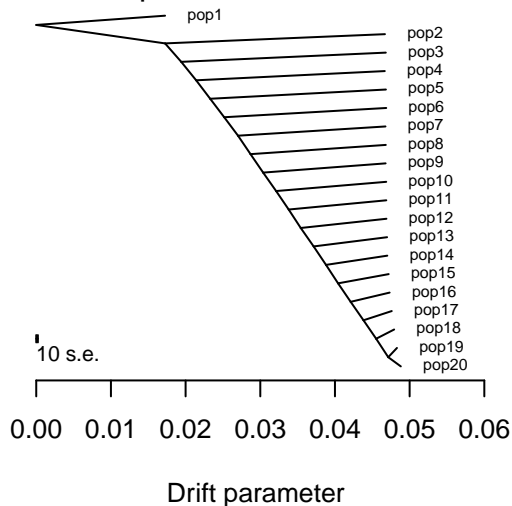

B. Residual fit from tree in A.

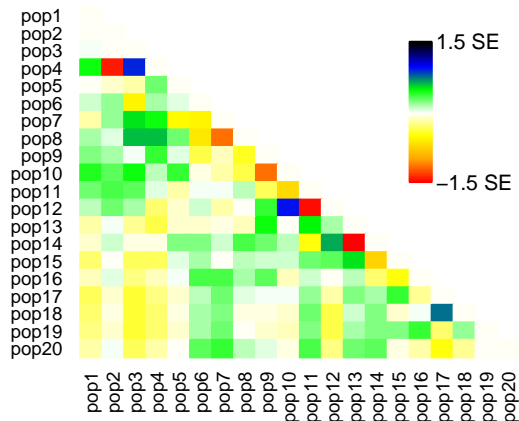

C. Example tree 2

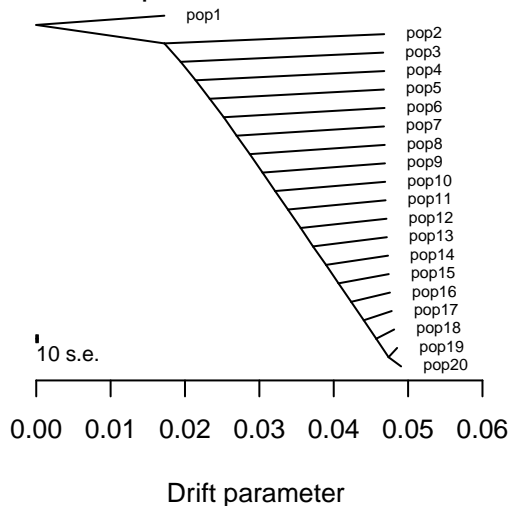

D. Residual fit from tree in C.

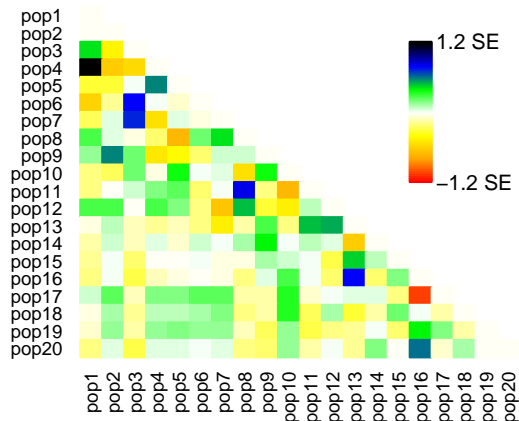

Supplement: Figure S6 — Example trees from simulations with long branches. We generated 100 tree-like datasets using the topology in Figure 2A in the main text, multiplying all branch lengths by 50. We then inferred the maximum likelihood tree. In Figure S5, we show the average inferred tree. Here, we show two representative trees (A. and C. and the residuals corresponding to each tree (B. and D.). (PDF) [file pgen.1002967.s006.pdf]

A. correct graph topology

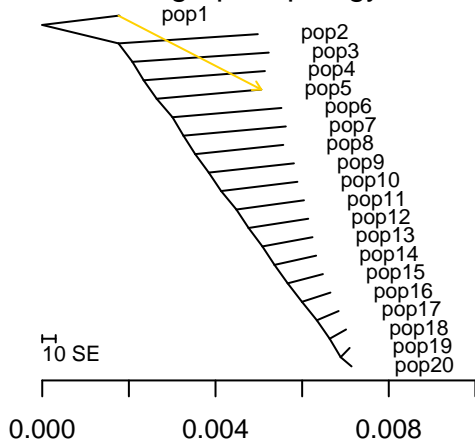

B. common error (w = 10%)

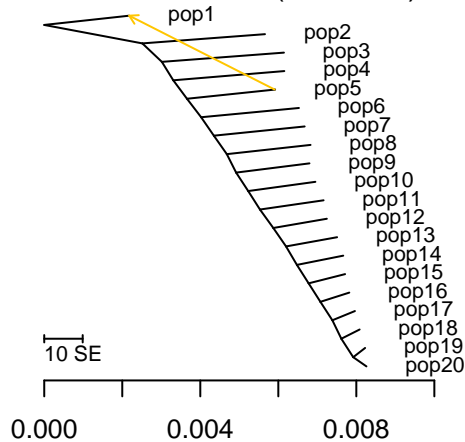

C. common error (w = 30%)

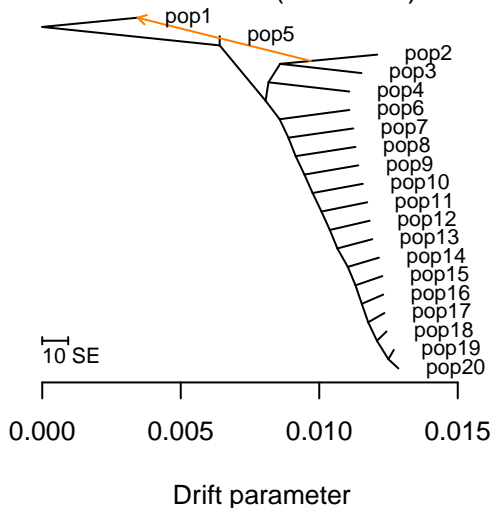

Supplement: Figure S7 — Representative errors in simulations. We examined the simulations in which TreeMix did not reach the correct answer. A. The correct topology for the simulations presented in the other panels. B. A representative example of an incorrect topology inferred from the simulations of a migration event with weight 10% from population 1 to population 5 (this topology accounted for all observed errors). C. A representative example of an incorrect topology inferred from the simulations of a migration event with weight 30% from population 1 to population 5 (this topology accounted for 95% of all errors). (PDF) [file pgen.1002967.s007.pdf]

A.

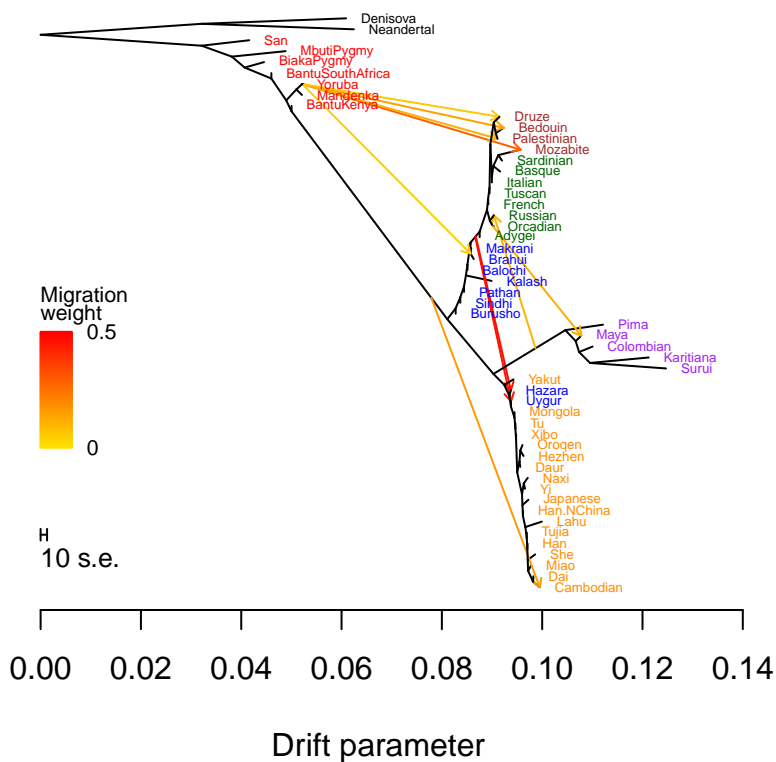

B.

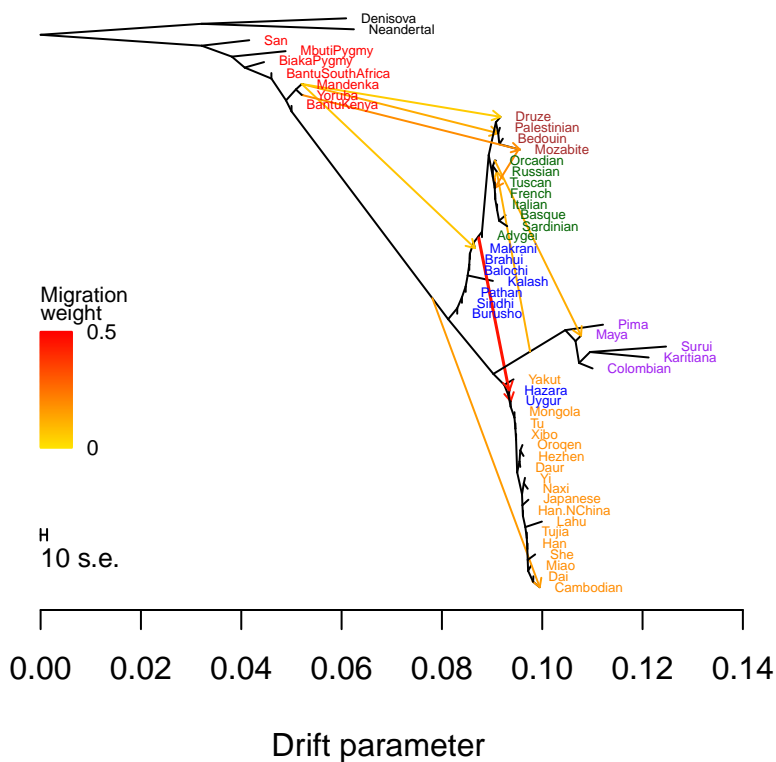

Supplement: Figure S8 — Replicate graphs inferred in the human data. These graphs were generated in an identical manner as Figure 4 in the main text, but using different random input orders for populations during tree-building. All random input orders gave very similar results. (PDF) [file pgen.1002967.s008.pdf]

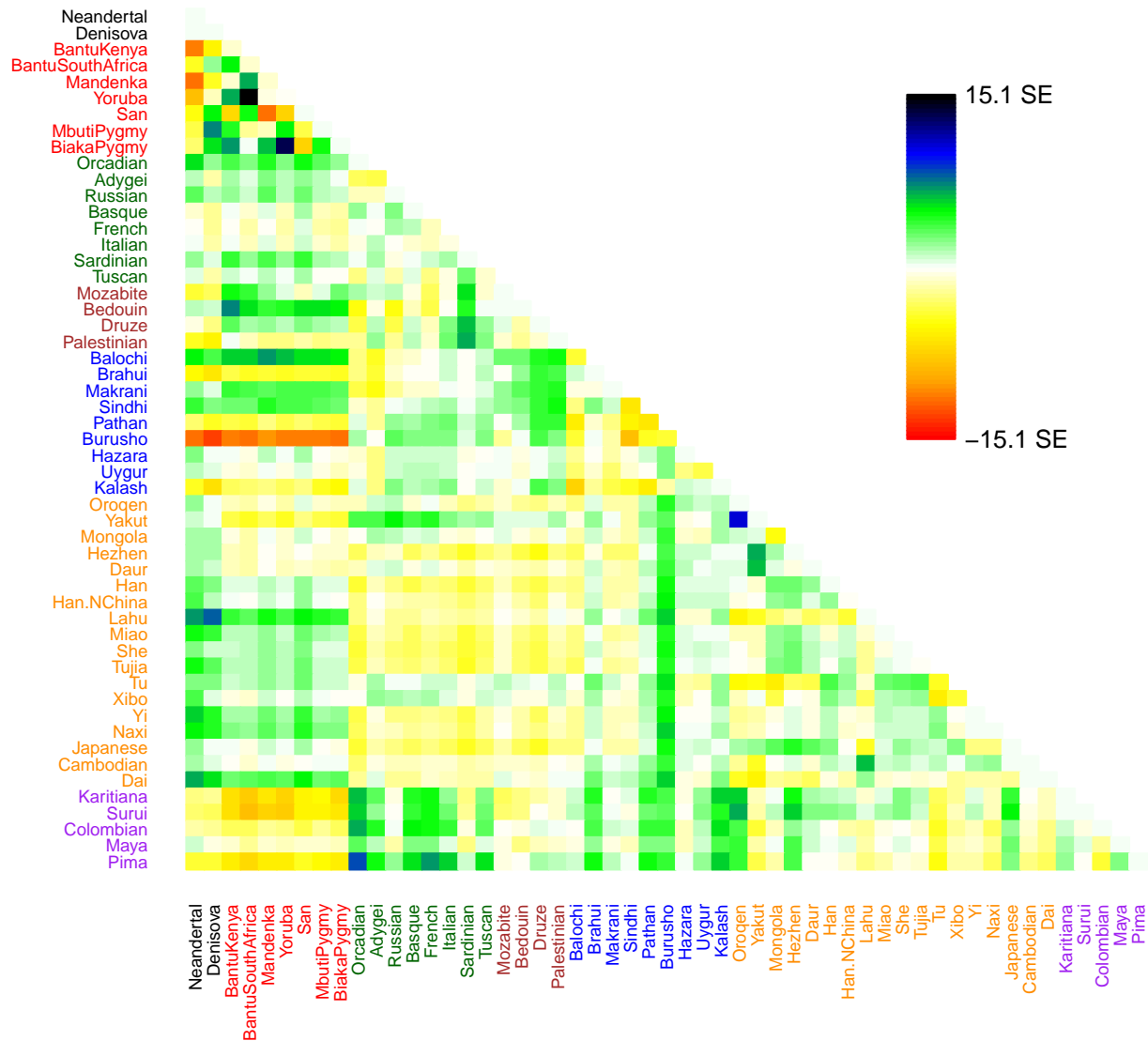

Supplement: Figure S9 — Residual fit from graph of human data presented in the main text. Plotted are the residuals from the fit of the graph presented in Figure 4 in the main text. (PDF) [file pgen.1002967.s009.pdf]

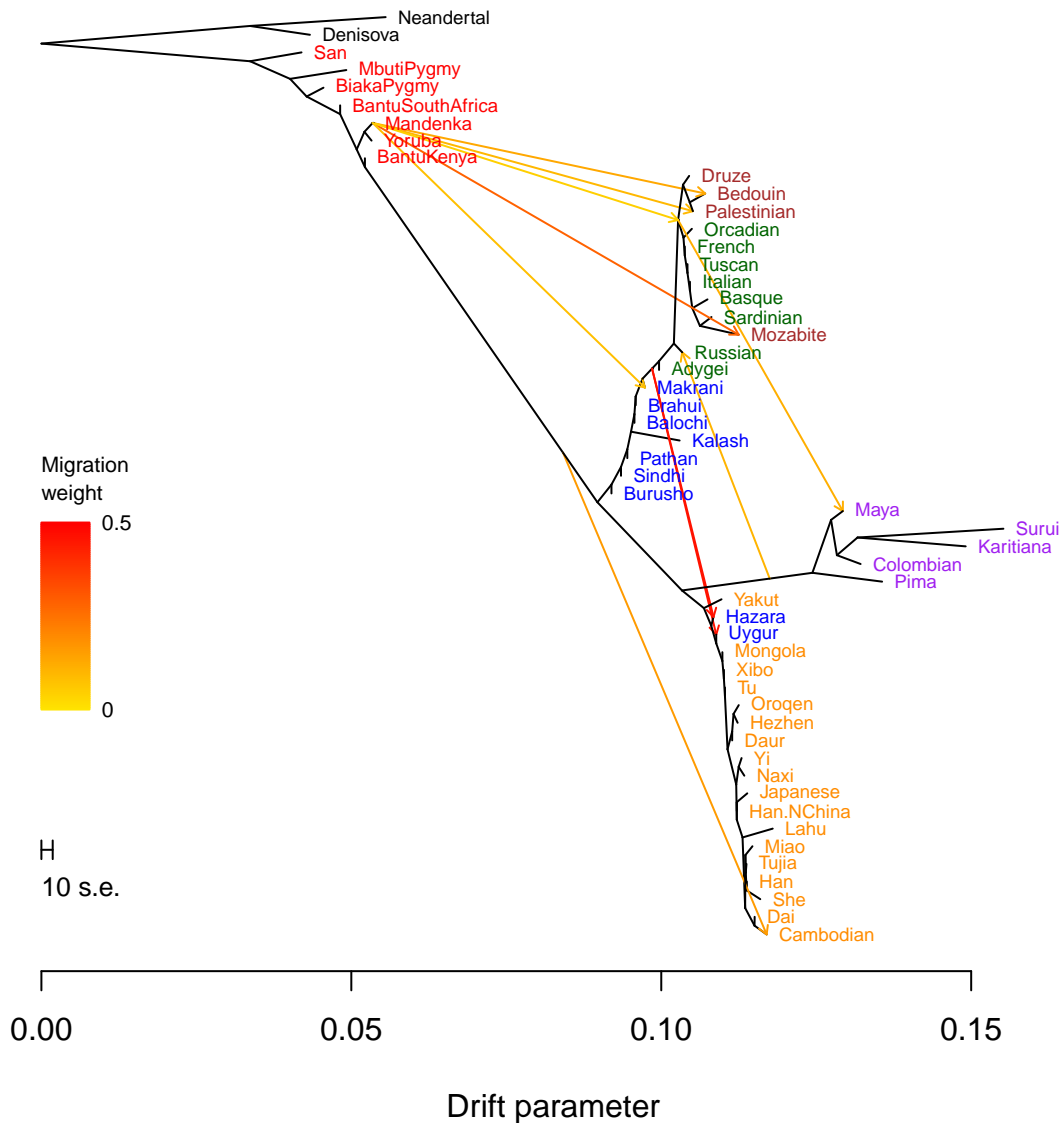

Supplement: Figure S10 — Graph inferred from SNPs ascertained in a single French individual. The graph was generated in an identical manner as Figure 4 in the main text, but using a panel of SNPs ascertained in a single French, rather than a single Yoruban, individual. The inferred graph is extremely similar to that in Figure 4. The one major difference is that, in this graph, the Mozabite appear as an admixture of a Sardinian population rather than a Middle Eastern population; this configuration is seen in some runs of TreeMix on the Yoruba-ascertained data (Figure S8A). (PDF) [file pgen.1002967.s010.pdf]

## A. Yoruba ascertainment

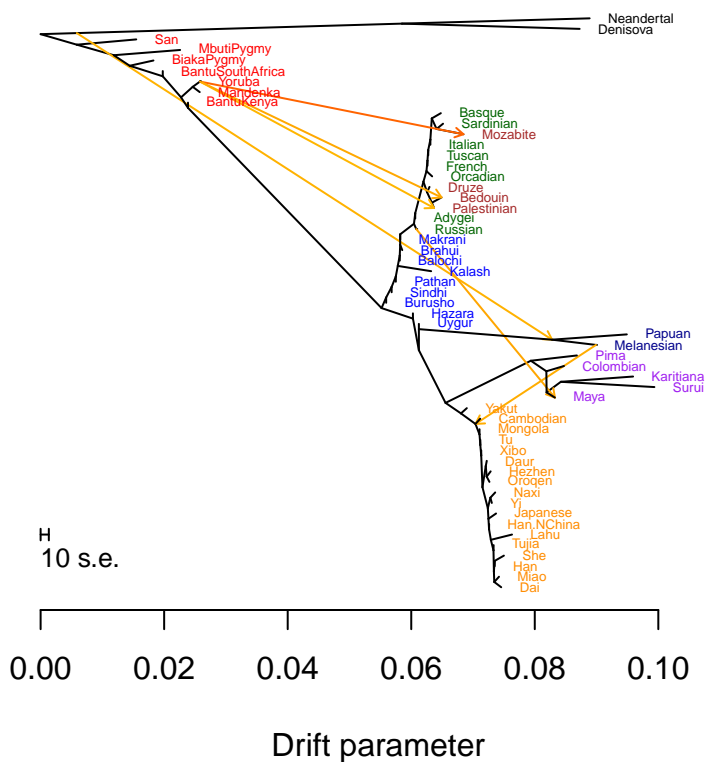

## B. French ascertainment

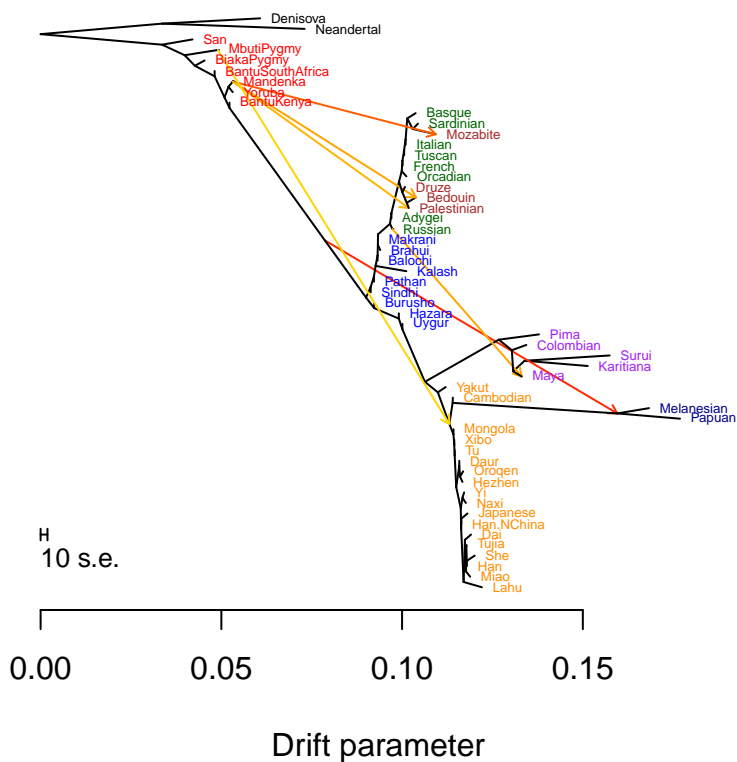

Supplement: Figure S12 — Graphs inferred using the human data including the Oceanians. We show the maximum likelihood graphs for the human data including the Oceanian populations, plotted in the same manner as in Figure 4 in the main text. Six migration edges were inferred in each graph. Graphs were inferred using the panel of SNPs ascertained in a single Yoruban individual (A.) and the panel of SNPs ascertained in a single French individual (B.). See Text S1 for discussion. (PDF) [file pgen.1002967.s012.pdf]

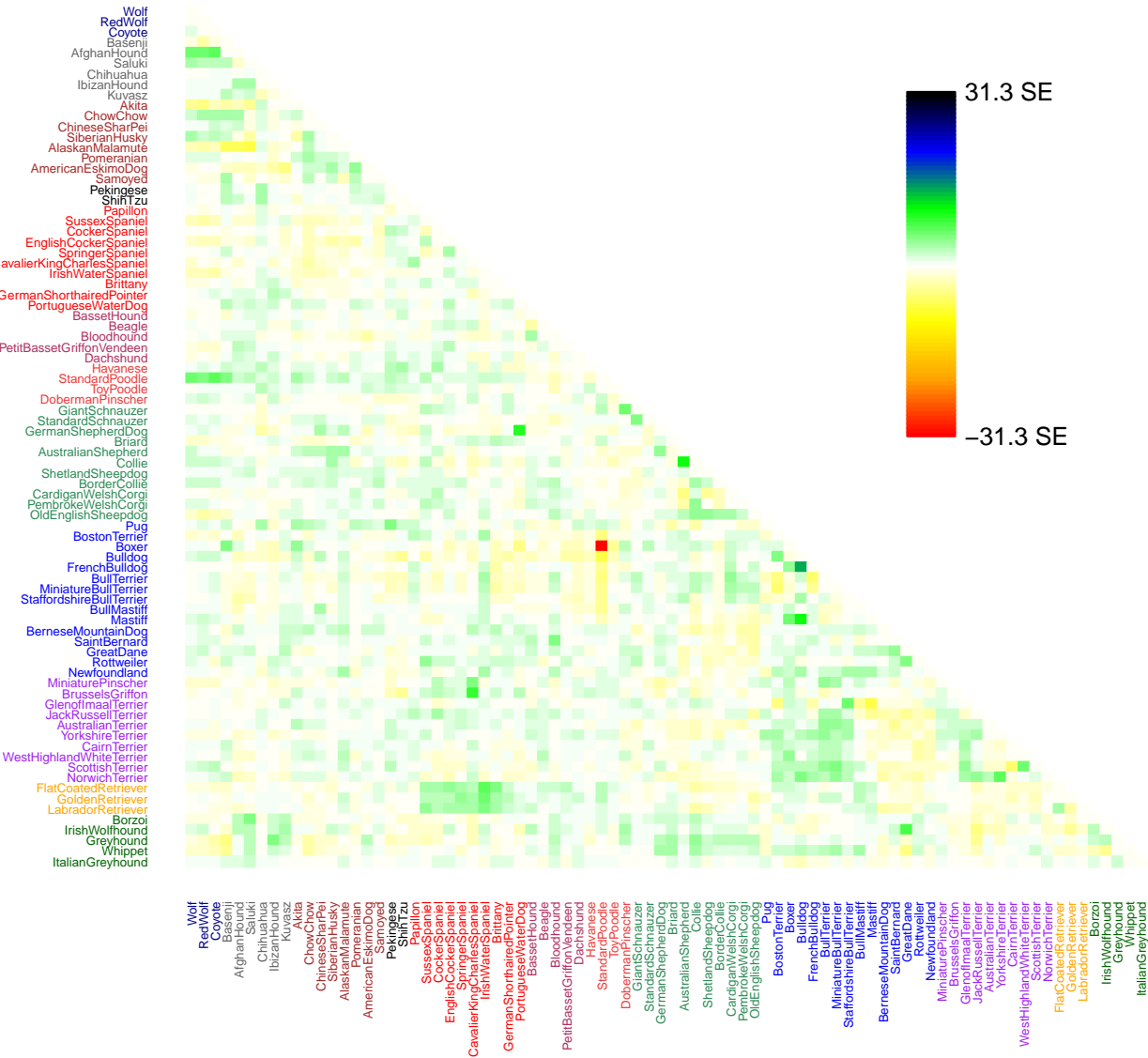

Supplement: Figure S13 — Residual fit from graph of dog data presented in the main text. Plotted are the residuals from the fit of the graph presented in Figure 6 in the main text. (PDF) [file pgen.1002967.s013.pdf]

A. Simulated grid

|      |      |       |
|------|------|-------|
| pop2 | pop3 | pop4  |
| pop5 | pop6 | pop7  |
| pop8 | pop9 | pop10 |

B. Example tree 1

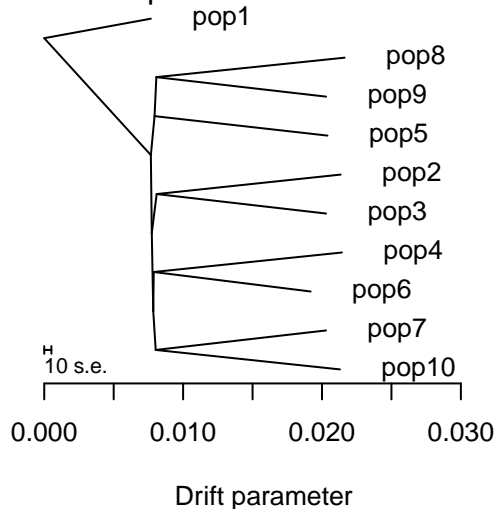

C. Example tree 2

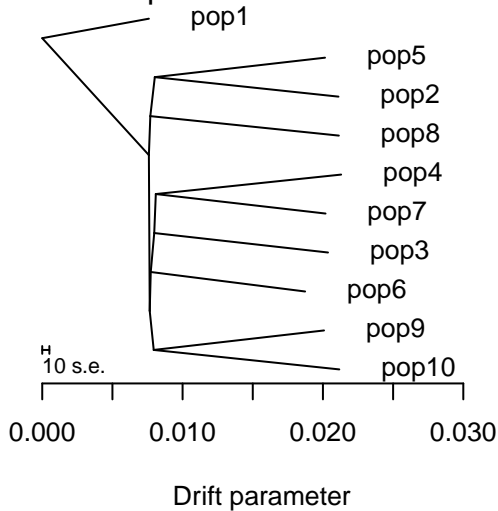

D. Example tree 3

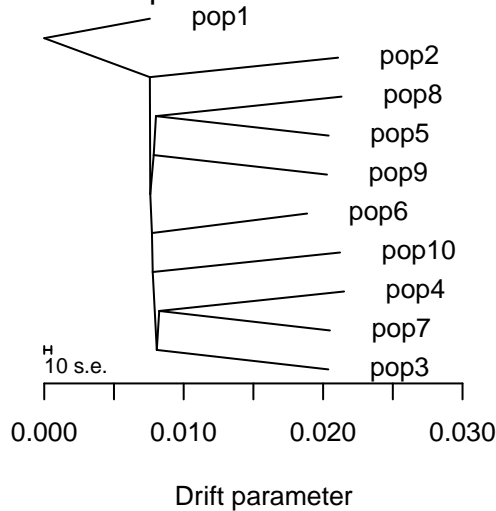

Supplement: Figure S14 — TreeMix run on populations with continuous migration. We simulated a set of populations on a lattice, where each population has constant gene flow at a rate of with neighboring populations. All populations split from an outgroup 16,400 generations in the past. The exact ms command is given in Text S1. The configuration of the lattice is presented in A. (population 1 is the outgroup). TreeMix inferred no consistent tree structure. Three representative trees are presented in B.-D. (PDF) [file pgen.1002967.s014.pdf]

A. Africa tree

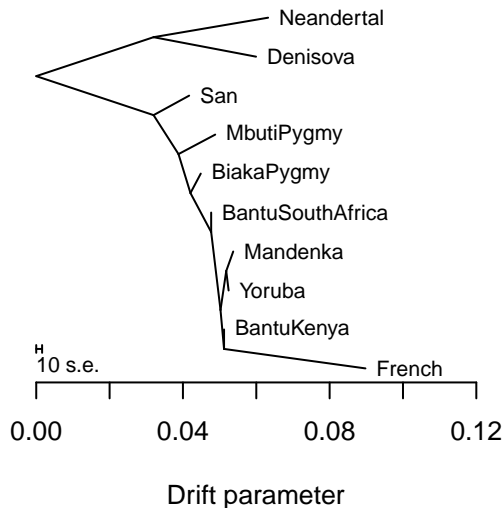

B. Africa tree residuals

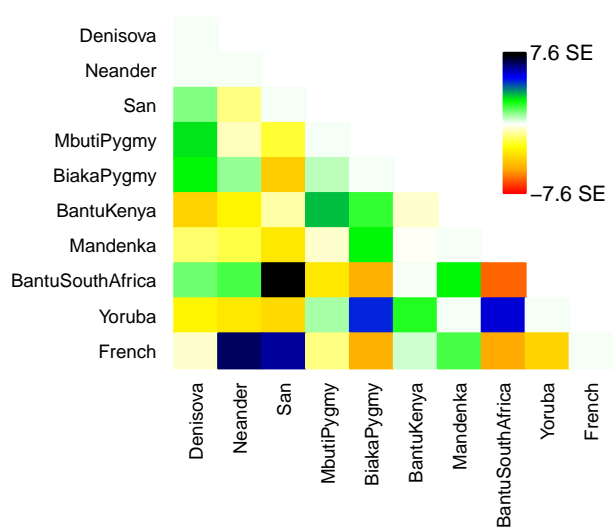

C. Africa graph

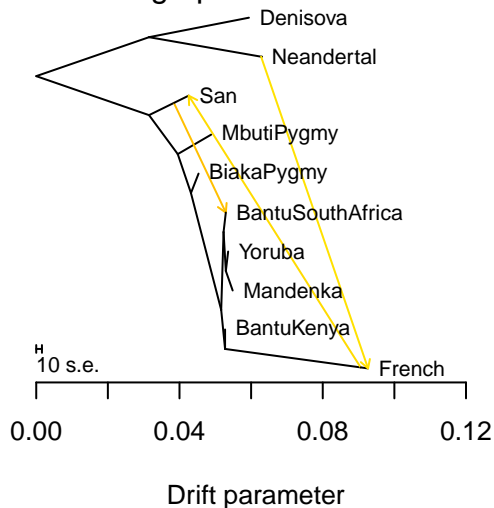

D. Africa graph residuals

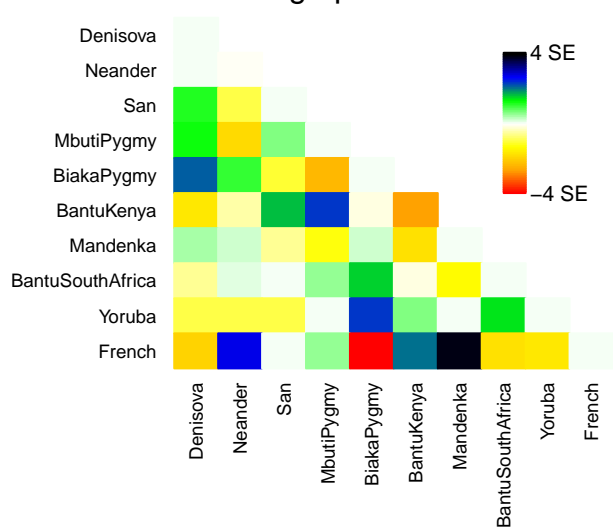

Supplement: Figure S15 — TreeMix run on human data using only a single non-African population. We inferred the maximum likelihood tree (A.) using only the African populations and one non-African population (French), using SNPs identified in a single Yoruban individual. In examining the residuals (B.), a relationship between the French and the Neandertal is clear. We then inferred three migration events (C.), where we do see that the French contain some Neandertal ancestry (). Residual fit for this graph is shown in D. (PDF) [file pgen.1002967.s015.pdf]
