# Supplementary material for: Inference of Population Splits and Mixtures from Genome-Wide Allele Frequency Data
Source: PLoS Genet. 2012 Nov 15;8(11):e1002967. doi: 10.1371/journal.pgen.1002967 (PMC3499260; doi:10.1371/journal.pgen.1002967)
Supplement: Figure S11 — Trees inferred using the human data including the Oceanians. We show the maximum likelihood trees and residuals for the human data including the Oceanian populations, plotted in the same manner as in Figure 3 in the main text. Trees were inferred using the panel of SNPs ascertained in a single Yoruban individual (A. and C.) and the panel of SNPs ascertained in a single French individual (B. and D.). (PDF) [file pgen.1002967.s011.pdf]

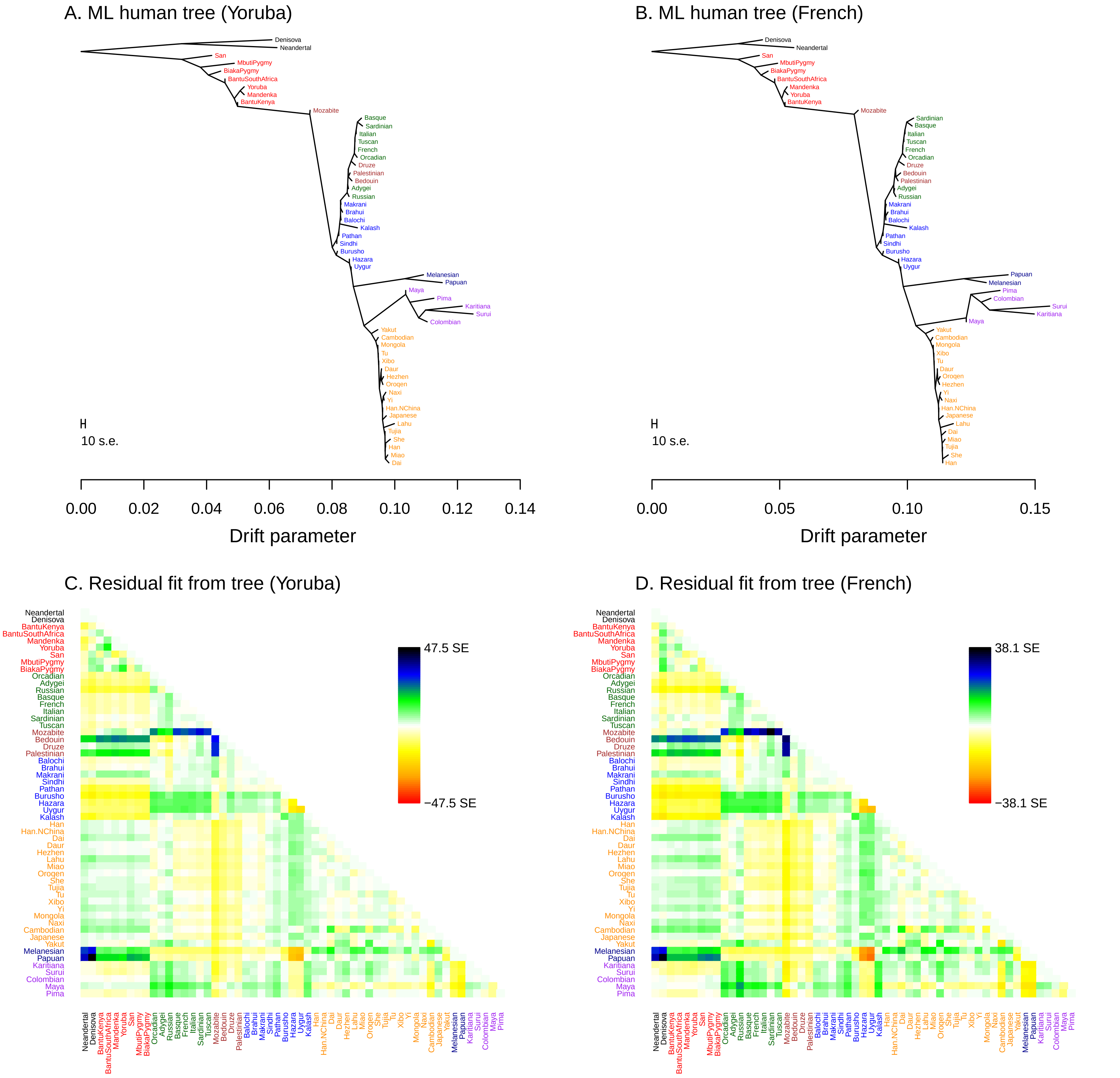

A. ML human tree (Yoruba)
Denisova
Neandertal
San
MbutiPygmy
BiakaPygmy
BantuSouthAfrica
Yoruba
Mandenka
BantuKenya
Mozabite
Basque
Sardinian
Italian
Tuscan
French
Orcadian
Druze
Palestinian
Bedouin
Adygei
Russian
Makrani
Brahui
Balochi
Kalash
Pathan
Sindhi
Burusho
Hazara
Uygur
Melanesian
Papuan
Maya
Pima
Karitiana
Surui
Colombian
Yakut
Cambodian
Mongola
Tu
Xibo
Daur
Hezhen
Oroqen
Naxi
Yi
Han.NChina
Japanese
Lahu
Tujia
She
Han
Miao
Dai
10 s.e.
0.00 0.02 0.04 0.06 0.08 0.10 0.12 0.14
Drift parameter
B. ML human tree (French)
Denisova
Neandertal
San
MbutiPygmy
BiakaPygmy
BantuSouthAfrica
Mandenka
Yoruba
BantuKenya
Mozabite
Sardinian
Basque
Italian
Tuscan
French
Orcadian
Druze
Bedouin
Palestinian
Adygei
Russian
Makrani
Brahui
Balochi
Kalash
Pathan
Sindhi
Burusho
Hazara
Uygur
Papuan
Melanesian
Pima
Colombian
Surui
Karitiana
Maya
Yakut
Cambodian
Mongola
Xibo
Tu
Daur
Oroqen
Hezhen
Yi
Naxi
Han.NChina
Japanese
Lahu
Dai
Miao
Tujia
She
Han
10 s.e.
0.00 0.05 0.10 0.15
Drift parameter
C. Residual fit from tree (Yoruba)
47.5 SE
−47.5 SE
Neandertal
Denisova
BantuKenya
BantuSouthAfrica
Mandenka
Yoruba
San
MbutiPygmy
BiakaPygmy
Orcadian
Adygei
Russian
Basque
French
Italian
Sardinian
Tuscan
Mozabite
Bedouin
Druze
Palestinian
Balochi
Brahui
Makrani
Sindhi
Pathan
Burusho
Hazara
Uygur
Kalash
Han
Han.NChina
Dai
Daur
Hezhen
Lahu
Miao
Orogen
She
Tujia
Tu
Xibo
Yi
Mongola
Naxi
Cambodian
Japanese
Yakut
Melanesian
Papuan
Karitiana
Surui
Colombian
Maya
Pima
D. Residual fit from tree (French)
38.1 SE
−38.1 SE
