# Supplementary material for: Inference of Population Splits and Mixtures from Genome-Wide Allele Frequency Data
Source: PLoS Genet. 2012 Nov 15;8(11):e1002967. doi: 10.1371/journal.pgen.1002967 (PMC3499260; doi:10.1371/journal.pgen.1002967)
Supplement: Text S1 — Supplementary Information. (PDF) [file pgen.1002967.s016.pdf]

Supplementary Material for: Inference of population splits and mixtures from genome-wide allele frequency data

Joseph K. Pickrell[1,3,†], Jonathan K. Pritchard[1,2,†]

[1] Department of Human Genetics and

[2] Howard Hughes Medical Institute, University of Chicago

[3] Current address: Department of Genetics, Harvard Medical School

† To whom correspondence should be addressed: joseph_pickrell@hms.harvard.edu, pritch@uchicago.edu

June 12, 2012

Correcting covariances for finite sample size. In the main text, we define the variance-covariance matrix $\hat{\mathbf{W}}$ of allele frequencies between populations without accounting for sampling variance. Here, we show the calculations corrected for sample size. Consider n biallelic loci typed in m populations of diploid individuals, and let the sample size in population i at locus k be N_{ik} (with missing data, the number of individuals can vary across loci). Let the counts of the two alleles in population i at locus k be n_{ik} and $2N_{ik} - n_{ik}$ (with one allele being arbitrarily defined as the reference in all that follows), the true allele frequency in the population be X_{ik}, and the observed allele frequency be $\hat{X}_{ik} = \frac{n_{ik}}{2N_{ik}}$. We assume the n_{ik} are binomially distributed with parameters $2N_{ik}$ and X_{ik}, and are independent for all i and k. Recall that the allele frequency in the ancestral population is x_A, and that the covariance between populations i and j with respect to the ancestral frequency x_A is $\mathbf{V}_{ij}$. We begin by defining $\mathbf{V}_{ij}$ using the observed allele frequencies at a single SNP k:

$$\begin{aligned}
\mathbf{V}_{ij} &= E[(\hat{X}_{ik} - x_A)(\hat{X}_{jk} - x_A)] &(1)\\
&= E\big[[(\hat{X}_{ik} - X_{ik}) + (X_{ik} - x_A)][(\hat{X}_{jk} - X_{jk}) + (X_{jk} - x_A)]\big] &(2)\\
&= E[(X_{ik} - x_A)(X_{jk} - x_A)] + E[(\hat{X}_{ik} - X_{ik})(\hat{X}_{jk} - X_{jk})]. &(3)
\end{aligned}$$

The bias in the estimate of $\mathbf{V}_{ij}$ is thus $E[(\hat{X}_{ik} - X_{ik})^2]$ if $i = j$ (i.e., it is the sampling variance in X_{ik}) and zero otherwise. This follows from the fact that the n_{ik} are assumed to be independent across i.

Now consider all n SNPs, and let the mean bias across all SNPs be B_i. At a given SNP k, the sampling variance in population j is $\hat{X}_{ik}$ is $\frac{X_{ik}(1-X_{ik})}{2N_{ik}}$ (from the binomial sampling of x_{ik}), so the mean bias across SNPs is proportional to $\overline{X_{ik}(1 - X_{ik})}$ (i.e., the mean across all SNPs of $X_{ik}(1 - X_{ik})$). A natural estimator of B_i is then:

$$B_i = \frac{h_i}{4N_i} \tag{4}$$

where h_i is an unbiased estimate of the heterozygosity in population i averaged over all SNPs [Nei, 1978]:

$$h_i = \frac{1}{n}\sum_{k=1}^{n} \frac{n_{ik}(2N_i - n_{ik})}{N_i(2N_i - 1)}. \tag{5}$$

As derived in the main text, the sample covariance of populations i and j, $\mathbf{W}_{ij}$, is:

$$\mathbf{W}_{ij} = \mathbf{V}_{ij} - \frac{1}{m}\sum_{k=1}^{m}\mathbf{V}_{ik} - \frac{1}{m}\sum_{k=1}^{m}\mathbf{V}_{jk} + \frac{1}{m^2}\sum_{k=1}^{m}\sum_{k'=1}^{m}\mathbf{V}_{kk'}. \tag{6}$$

The bias in the estimate of $\hat{\mathbf{W}}_{ij}$ (let us call this B'_{ij}) is then:

$$B'_{ij} = I_{[i=j]}B_i - \frac{B_i}{m} - \frac{B_j}{m} + \frac{\sum_{k=1}^{m} B_k}{m^2} \tag{7}$$

where $I_{[i=j]}$ is an indicator that evaluates to 1 if $i = j$ and zero otherwise. We can then estimate the unbiased covariance $\hat{\mathbf{W}}_{ij}$ as:

$$\hat{\mathbf{W}}_{ij} = \frac{\sum_{k=1}^{n}(\hat{X}_{ik} - \mu_k)(\hat{X}_{jk} - \mu_k)}{n} - B'_{ij} \tag{8}$$

where $\mu_k = \frac{\sum_{i=1}^{m} \hat{X}_{ik}}{m}$. If there is missing data in either population i or population j, we simply ignore the SNP for that pairwise comparison of populations. Since the mean allele frequency across populations is important here, large amounts of missing data (or correlated missingness between populations) could result in skewed covariances. We thus exclude populations with large amounts of missing data.

Nonidentifiability of the drift parameters in an admixed population. In the main text, we write down a model for the allele frequencies in an admixed population, and claim that the amount of genetic drift occurring before and after the mixture event are nonidentifiable. Consider the graph in Supplementary Figure 1. We can write down the expected variances and covariances involving the admixed population:

$$\mathbf{V}_{12} = (1 - w)c_4 x_A[1 - x_A] \tag{9}$$

$$\mathbf{V}_{23} = wc_5 x_A[1 - x_A] \tag{10}$$

$$\mathbf{V}_{22} = [c_1 + w^2(c_2 + c_5) + (1 - w)^2(c_3 + c4)]x_A[1 - x_A] \tag{11}$$

and we are interested in estimating w, c_1, c_2, and c_3. It is clear from the above that c_1, c_2, and c_3 do not appear except as a linear combination. Adding additional populations does not add additional information about these parameters, unless they are assumed to result from the same mixture event.

We choose to set c_2 and c_1 to zero, and estimate only c_3, which can now be thought of as a composite branch length that sums all the three components of genetic drift. A subtle point is that all of this drift is weighted by $(1 - w)$. When estimating w, then, the true relative contributions of c_1, c_2, and c_3 could lead to a bias in the estimation of w. For example, if c_1 and/or c_2 are large, this could bias the estimation of $(1 - w)$ upwards. We believe this is likely the cause of the downward bias in w in the simulations in Figure 2D in the main text.

Graph representation of the *TreeMix* model. In the main text, we describe a specification of $\mathbf{V}$ (the variance/covariance matrix of allele frequencies, defined with respect to an ancestral population) in terms of a system of linear equations. A useful alternate notation describes $\mathbf{V}$ in terms of a graph [Koller and Friedman, 2009]. Let G be a rooted, directed, acyclic graph with a set of nodes N and a set of directed edges E. Each edge e has an associated length, c_e, and a weight, w_e (between zero and one). A special class of edges, called migration edges, are forced to have length zero. The sum of weights of edges entering a given node is one. There is one node which is the root (a node with only outgoing edges), and each population corresponds to a tip (a node with

only incoming edges).

Define $\{P_i\}$ to be the set of all possible paths in G from the root to the tip corresponding to population i (if the graph is a tree, there is only one such path). Each individual path p has a weight, $w(p) = \Pi_{e \in p} w_e$. Now define the overlap between two paths as:

$$O(p_i, p_j) = \sum_{e \in p_i} w(p_i) w(p_j) I[e \in p_j] c_e \tag{12}$$

where $I[e \in p_j]$ is a function that evaluates to one if edge e is in p_j, and zero otherwise. We can now write down the expected covariance between populations i and j as:

$$\mathbf{V}_{ij} = \sum_{p_i \in \{P_i\}} \sum_{p_j \in \{P_j\}} O(p_i, p_j). \tag{13}$$

In the special case where G is a tree, there is only one path per population and all of the edges have weight one, and so $\mathbf{V}_{ij}$ reduces to a sum of the lengths of branches shared by the two populations.

Relationship of this model to $f-$ statistics. Tests for "treeness" in three and four-population trees [Keinan et al., 2007; Reich et al., 2009] have used a framework in which the distances between populations are quantified in terms of "$f-$statistics" comparing the allele frequencies between the populations. Below, we briefly describe these tests in the notation of our model. Consider the expected f_3 statistic calculated between populations 1, 2, and 3, with corresponding allele frequencies X_1, X_2, and X_3.

$$f_3(X_1; X_2, X_3) = E[(X_1 - X_2)(X_1 - X_3)] \tag{14}$$

$$= E\big[[(X_1 - x_A) - (X_2 - x_A)][(X_1 - x_A) - (X_3 - x_A)]\big] \tag{15}$$

$$= \mathbf{V}_{11} - \mathbf{V}_{12} - \mathbf{V}_{13} + \mathbf{V}_{23}. \tag{16}$$

Consider the situation where populations 1 and 3 form a clade relative to 2 (i.e., population 2 is an outgroup). If population X_1 is not admixed, this reduces to:

$$f_3(X_1; X_2, X_3) = \mathbf{V}_{11} - \mathbf{V}_{13}. \tag{17}$$

This is necessarily greater than zero (since $\mathbf{V}_{13} <= \mathbf{V}_{11}$). If X_1 is admixed, then $\mathbf{V}_{12}$ can be important and the f_3 statistic can be negative. A test for a negative f_3 statistic is thus a test for admixture in population X_1 [Reich et al., 2009]. However, this signal can be weakened by large amounts of drift in X_1 (i.e., a large $\mathbf{V}_{11}$), or mixture between X_2 and X_3 [Reich et al., 2009].

Similarly, consider the expected f_4 statistic computed on the tree [[1,2],[3,4]], where 1, 2, 3, and

4 are populations, and X_1, X_2, X_3 and X_4 are the corresponding allele frequencies:

$$\begin{aligned} f_4(X_1, X_2; X_3, X_4) &= E[(X_1 - X_2)(X_3 - X_4)] &(18)\\ &= E\big[[(X_1 - x_A) - (X_2 - x_A)][(X_3 - x_A) - (X_4 - x_A)]\big] &(19)\\ &= \mathbf{V}_{13} - \mathbf{V}_{23} - \mathbf{V}_{14} + \mathbf{V}_{24} &(20)\\ & &(21)\end{aligned}$$

If the tree is correct (i.e., if populations 1 and 2 are a clade relative to populations 3 and 4), all of these quantities are zero. A test for a non-zero f_4 statistic is thus a test for treeness [Reich et al., 2009].

Simulation commands. For all simulations, we used *ms* [Hudson, 2002]. To generate the tree-like data depicted in Figure 2A in the main text, the command is:

```
ms 400 400 -t 200 -r 200 500000 -I 20 20 20 20 20 20 20 20 20 20 20 20 20 20 20 20
20 20 20 20 20 -en 0.00270 20 0.025 -ej 0.00275 20 19 -en 0.00545 19 0.025 -ej 0.00550
19 18 -en 0.00820 18 0.025 -ej 0.00825 18 17 -en 0.01095 17 0.025 -ej 0.011 17 16 -en
0.01370 16 0.025 -ej 0.01375 16 15 -en 0.01645 15 0.025 -ej 0.01650 15 14 -en 0.01920
14 0.025 -ej 0.01925 14 13 -en 0.02195 13 0.025 -ej 0.02200 13 12 -en 0.02470 12 0.025
-ej 0.02475 12 11 -en 0.02745 11 0.025 -ej 0.02750 11 10 -en 0.03020 10 0.025 -ej 0.03025
10 9 -en 0.03295 9 0.025 -ej 0.03300 9 8 -en 0.03570 8 0.025 -ej 0.03575 8 7 -en 0.03845
7 0.025 -ej 0.03850 7 6 -en 0.04120 6 0.025 -ej 0.04125 6 5 -en 0.04395 5 0.025 -ej
0.04400 5 4 -en 0.04670 4 0.025 -ej 0.04675 4 3 -en 0.04945 3 0.025 -ej 0.04950 3 2
-en 0.05220 2 0.025 -ej 0.05225 2 1
```

To create trees with considerably longer branch lengths, we multiplied all branch lengths by 50:

```
ms 400 400 -t 200 -r 200 500000 -I 20 20 20 20 20 20 20 20 20 20 20 20 20 20 20 20
20 20 20 20 20 -en 0.135 20 0.025 -ej 0.1375 20 19 -en 0.2725 19 0.025 -ej 0.275 19
18 -en 0.41 18 0.025 -ej 0.4125 18 17 -en 0.5475 17 0.025 -ej 0.55 17 16 -en 0.685
16 0.025 -ej 0.6875 16 15 -en 0.8225 15 0.025 -ej 0.825 15 14 -en 0.96 14 0.025 -ej
0.9625 14 13 -en 1.0975 13 0.025 -ej 1.1 13 12 -en 1.235 12 0.025 -ej 1.2375 12 11
-en 1.3725 11 0.025 -ej 1.375 11 10 -en 1.51 10 0.025 -ej 1.5125 10 9 -en 1.6475 9
0.025 -ej 1.65 9 8 -en 1.785 8 0.025 -ej 1.7875 8 7 -en 1.9225 7 0.025 -ej 1.925 7
6 -en 2.06 6 0.025 -ej 2.0625 6 5 -en 2.1975 5 0.025 -ej 2.2 5 4 -en 2.335 4 0.025
-ej 2.3375 4 3 -en 2.4725 3 0.025 -ej 2.475 3 2 -en 2.61 2 0.025 -ej 2.6125 2 1
```

For simulations with migration, we added a migration event approximately 100 generations before the present. For example, migration from population 1 to population 10:

```
ms 400 400 -t 200 -r 200 500000 -I 20 20 20 20 20 20 20 20 20 20 20 20 20 20 20 20
```

```
20 20 20 20 20 -em 0.002675 10 1 4000 -en 0.00270 20 0.025 -em 0.00270 10 1 0 -ej 0.00275
20 19 -en 0.00545 19 0.025 -ej 0.00550 19 18 -en 0.00820 18 0.025 -ej 0.00825 18 17
-en 0.01095 17 0.025 -ej 0.011 17 16 -en 0.01370 16 0.025 -ej 0.01375 16 15 -en 0.01645
15 0.025 -ej 0.01650 15 14 -en 0.01920 14 0.025 -ej 0.01925 14 13 -en 0.02195 13 0.025
-ej 0.02200 13 12 -en 0.02470 12 0.025 -ej 0.02475 12 11 -en 0.02745 11 0.025 -ej 0.02750
11 10 -en 0.03020 10 0.025 -ej 0.03025 10 9 -en 0.03295 9 0.025 -ej 0.03300 9 8 -en
0.03570 8 0.025 -ej 0.03575 8 7 -en 0.03845 7 0.025 -ej 0.03850 7 6 -en 0.04120 6 0.025
-ej 0.04125 6 5 -en 0.04395 5 0.025 -ej 0.04400 5 4 -en 0.04670 4 0.025 -ej 0.04675
4 3 -en 0.04945 3 0.025 -ej 0.04950 3 2 -en 0.05220 2 0.025 -ej 0.05225 2 1
```

For simulations of populations exchanging migrants on a lattice, we used the following command:

```
ms 200 1500 -t 40 -r 40 100000 -I 10 20 20 20 20 20 20 20 20 20 20 -ma x 0 0 0 0 0
0 0 0 0 0 x 0.1 0 0.1 0.1 0 0 0 0 0 0.1 x 0.1 0.1 0.1 0.1 0 0 0 0 0 0.1 x 0 0.1 0.1
0 0 0 0 0.1 0.1 0 x 0.1 0 0.1 0.1 0 0 0.1 0.1 0.1 0.1 x 0.1 0.1 0.1 0.1 0 0 0.1 0.1
0 0.1 x 0 0.1 0.1 0 0 0 0 0.1 0.1 0 x 0.1 0 0 0 0 0 0.1 0.1 0.1 0.1 x 0.1 0 0 0 0 0
0.1 0.1 0 0.1 x -ej 0.41 10 1 -ej 0.41 9 1 -ej 0.41 8 1 -ej 0.41 7 1 -ej 0.41 6 1 -ej
0.41 5 1 -ej 0.41 4 1 -ej 0.41 3 1 -ej 0.41 2 1
```

Discussion of simulation errors. In Figure 2C in the main text, we showed that *TreeMix* was extremely accurate in most simulation situations. However, there are a few situations in which it performed poorly. Most notably, this was for simulated admixture between population 1 and 5. The errors in these simulations tended to be of the same type (Supplementary Figure 7). Additionally, in the simulations of migration from population 15 to population 20 with a weight of 10%, there was also a considerable error rate. However, these errors were not consistent across simulations, and are likely due to the algorithm simply not detecting the admixture event at all.

Analysis of human data including Oceanian populations. As described in the main text, in the human HGDP data we used two sets of allele frequencies with different ascertainment schemes– one at SNPs ascertained by sequencing a single Yoruban individual, and one at SNPs ascertained by sequencing a single French individual. We initially ran *TreeMix* on both data sets using all populations to estimate the maximum likelihood trees. The trees estimated using the two ascertainment schemes are nearly identical (Supplementary Figure 11. We then used *TreeMix* to identify migration events. The algorithm arrived at quite different conclusions about the Oceanian populations in the two different data sets (recall that these are the exact same individuals, just genotyped at different SNPs) (Supplementary Figure 15). In the Yoruba-ascertained data, the East Asian populations are inferred to be admixed, with the Melanesians as a source population. However, in the French-ascertained data, the Oceanians are inferred to be admixed. When the Oceanian

populations are excluded from analysis, the algorithm comes up with nearly the same graph in both datasets (Figure 4 in the main text and Supplementary Figure 10).

It is not immediately clear why there is a discrepancy between these two datasets when looking at Oceanian populations. However, Oceania has a particularly complicated genetic makeup, involving at least four distinct components of ancestry: Denisovan gene flow, Neandertal gene flow, native Oceanian, and gene flow from Austronesian speakers [Reich et al., 2010, 2011; Wollstein et al., 2010]. These different components of ancestry may be picked up to differing extents by SNPs from the different ascertainment panels, leading to conflicting results.

List of migration events inferred in the human data. Here we list the ten migration edges inferred in the human data and present in Figure 4 in the main text:

1. Yoruba → Mozabite, $w = 19\%$
2. Mandenka → (Palestinian,(Bedouin, Mozabite)), $w = 13\%$
3. Mandenka → Druze, $w = 6\%$
4. Mankenka → (Brahui, Makrani), $w = 6\%$
5. Ancestral non-African → Cambodian, $w = 17\%$
6. (All Europe, Middle East) → Hazara, $w = 46\%$
7. (All Europe, Middle East) → Uygur, $w = 46\%$
8. All Native Americans → Russian, $w = 12\%$
9. (Tuscan(French(Italian,(Basque,Sardinian) → Maya, $w = 12\%$
10. Mozabite → (Tuscan(French(Italian,(Basque,Sardinian), $w = 22\%$

List of migration events inferred in the dog data. Here we list the ten migration edges inferred in the dog data and present in Figure 6 in the main text:

1. (Greyhound, Whippet) → Borzoi, $w = 47\%$
2. (AlaskanMalamute,SiberianHusky) → Samoyed, $w = 47\%$
3. Wolf → Basenji, $w = 25\%$
4. (ChowChow,ChineseSharPei) → (Pekingese, Shih Tzu), $w = 28\%$
5. Bulldog → Bull mastiff, $w = 31\%$
6. Boxer → Chinese shar-pei, $w = 8\%$
7. Siberian Husky → (Akita,(ChowChow,ChineseSharPei)) $w = 17\%$

8. Wolf $\rightarrow$ Boxer, $w = 8\%$
9. Mastiff$\rightarrow$ Saint Bernard, $w = 13\%$
10. Whippet $\rightarrow$ Italian Greyhound, $w = 37\%$

References

Hudson, R. R., 2002. Generating samples under a Wright-Fisher neutral model of genetic variation. *Bioinformatics*, **18**(2):337–8.

Keinan, A., Mullikin, J. C., Patterson, N., and Reich, D., 2007. Measurement of the human allele frequency spectrum demonstrates greater genetic drift in East Asians than in Europeans. *Nat Genet*, **39**(10):1251–5.

Koller, D. and Friedman, N., 2009. *Probabilistic graphical models: principles and techniques*. The MIT Press.

Nei, M., 1978. Estimation of average heterozygosity and genetic distance from a small number of individuals. *Genetics*, **89**(3):583–90.

Reich, D., Green, R. E., Kircher, M., Krause, J., Patterson, N., Durand, E. Y., Viola, B., Briggs, A. W., Stenzel, U., Johnson, P. L. F., *et al.*, 2010. Genetic history of an archaic hominin group from Denisova Cave in Siberia. *Nature*, **468**(7327):1053–60.

Reich, D., Patterson, N., Kircher, M., Delfin, F., Nandineni, M. R., Pugach, I., Ko, A. M.-S., Ko, Y.-C., Jinam, T. A., Phipps, M. E., *et al.*, 2011. Denisova admixture and the first modern human dispersals into Southeast Asia and Oceania. *Am J Hum Genet*, **89**(4):516–28.

Reich, D., Thangaraj, K., Patterson, N., Price, A. L., and Singh, L., 2009. Reconstructing Indian population history. *Nature*, **461**(7263):489–94.

Wollstein, A., Lao, O., Becker, C., Brauer, S., Trent, R. J., Nürnberg, P., Stoneking, M., and Kayser, M., 2010. Demographic history of Oceania inferred from genome-wide data. *Curr Biol*, **20**(22):1983–92.
